# Environmental filtering predicts plant‐community trait distribution and diversity: Kettle holes as models of meta‐community systems

**DOI:** 10.1002/ece3.4883

**Published:** 2019-01-21

**Authors:** Sissi Lozada‐Gobilard, Susanne Stang, Karin Pirhofer‐Walzl, Thomas Kalettka, Thilo Heinken, Boris Schröder, Jana Eccard, Jasmin Joshi

**Affiliations:** ^1^ Biodiversity Research/Systematic Botany University of Potsdam Potsdam Germany; ^2^ Berlin‐Brandenburg Institute of Advanced Biodiversity Research (BBIB) Berlin Germany; ^3^ Plant Ecology, Institut fuer Biologie Freie Universitaet Berlin Berlin Germany; ^4^ Leibniz‐Centre for Agricultural Landscape Research (ZALF) Müncheberg Germany; ^5^ General Botany University of Potsdam Potsdam Germany; ^6^ Landscape Ecology and Environmental Systems Analysis, Institute of Geoecology Technische Universität Braunschweig Braunschweig Germany; ^7^ Animal Ecology University of Potsdam Potsdam Germany; ^8^ Institute for Landscape and Open Space Hochschule für Technik HSR Rapperswil Rapperswil Switzerland

**Keywords:** biodiversity, dispersal, disturbance, landscape diversity, life‐history traits, plant diversity, seed bank, species assembly, wetland vegetation

## Abstract

Meta‐communities of habitat islands may be essential to maintain biodiversity in anthropogenic landscapes allowing rescue effects in local habitat patches. To understand the species‐assembly mechanisms and dynamics of such ecosystems, it is important to test how local plant‐community diversity and composition is affected by spatial isolation and hence by dispersal limitation and local environmental conditions acting as filters for local species sorting.We used a system of 46 small wetlands (kettle holes)—natural small‐scale freshwater habitats rarely considered in nature conservation policies—embedded in an intensively managed agricultural matrix in northern Germany. We compared two types of kettle holes with distinct topographies (flat‐sloped, ephemeral, frequently plowed kettle holes vs. steep‐sloped, more permanent ones) and determined 254 vascular plant species within these ecosystems, as well as plant functional traits and nearest neighbor distances to other kettle holes.Differences in alpha and beta diversity between steep permanent compared with ephemeral flat kettle holes were mainly explained by species sorting and niche processes and mass effect processes in ephemeral flat kettle holes. The plant‐community composition as well as the community trait distribution in terms of life span, breeding system, dispersal ability, and longevity of seed banks significantly differed between the two habitat types. Flat ephemeral kettle holes held a higher percentage of non‐perennial plants with a more persistent seed bank, less obligate outbreeders and more species with seed dispersal abilities via animal vectors compared with steep‐sloped, more permanent kettle holes that had a higher percentage of wind‐dispersed species. In the flat kettle holes, plant‐species richness was negatively correlated with the degree of isolation, whereas no such pattern was found for the permanent kettle holes.Synthesis: Environment acts as filter shaping plant diversity (alpha and beta) and plant‐community trait distribution between steep permanent compared with ephemeral flat kettle holes supporting species sorting and niche mechanisms as expected, but we identified a mass effect in ephemeral kettle holes only. Flat ephemeral kettle holes can be regarded as meta‐ecosystems that strongly depend on seed dispersal and recruitment from a seed bank, whereas neighboring permanent kettle holes have a more stable local species diversity.

## 1 INTRODUCTION

A meta‐community has been defined as “set of local communities that are linked by dispersal of multiple potentially interacting species” (Leibold et al., 2004). Local community assembly within a meta‐community is therefore influenced by local interactions and regional processes (Logue, Mouquet, Peter, & Hillebrand, 2011; Wilson, 1992). This interdependence of interactions and processes has been classified into four paradigms by Leibold et al. (2004) based on species characteristics (mainly dispersal) and environmental conditions: Species Sorting (SS), Mass Effects (ME), Patch Dynamics (PD), and Neutral Model (NM) (revised by Logue et al., 2011). In two of these processes, Species Sorting (SS) and Mass Effects (ME), environmental heterogeneity plays a role in filtering species due to niche difference (in case of SS) or due to a source–sink mechanism when patches are interconnected (in case of ME).

Environmental filtering is based on the idea that abiotic factors select species with particular traits and phenotypes to establish, persist, and reproduce (environmental filtering sensu stricto), but establishment and persistence of species also depend on biotic interactions (Bartelt‐Ryser, Joshi, Schmid, Brandl, & Balser, 2005; Kraft et al., 2015). Indeed, studies focusing on local–regional environmental gradients (Butterfield & Munson, 2016; Laliberte, Zemunik, & Turner, 2014) and (few) at global scale (e.g., Henriques‐Silva, Lindo, & Peres‐Neto, 2013; Le Bagousse‐Pinguet et al., 2017) concluded that it is very difficult to separate biotic interactions from environmental filtering sensu stricto. In addition, identification of relevant environmental filters strongly depends on the selected scale (Münkemüller et al., 2014).

When organisms move under a meta‐community framework, they connect habitats modifying the flow of resources and consequently the entire functioning of the ecosystem (Gounand, Harvey, Little, & Altermatt, 2018). On this basis, the concept of “meta‐ecosystem” was proposed by Loreau, Mouquet, and Holt (2003). These authors defined a meta‐ecosystem as a set of ecosystems connected by spatial flows of energy, materials, and organisms across ecosystem boundaries. In this meta‐ecosystem dynamic, different types of movements or processes (e.g., predation, biomass recycling, mating aggregations) are involved in the coupling of ecosystems (Gounand et al., 2018), but empirical data on the movement ecology on relevant spatial scales for meta‐community couplings are still limited.

In plant communities, passive movement has mainly been studied in seed dispersal (e.g., Figuerola & Green, 2002; Soons, Brochet, Kleyheeg, & Green, 2016) and less often through pollen despite its equal importance, for example, for invasive species (Harmon‐Threatt, Burns, Shemyakina, & Knight, 2009). Studies using genetic techniques to track both pollen and seed dispersal have successfully assessed functional connectivity of plant populations (Aavik, Holderegger, Edwards, & Billeter, 2013) highlighting the importance of both dispersal processes. In addition, features such asexual reproduction (clonality), extreme longevity (trees, clonal plants), or the ability to survive under unfavorable conditions (seed bank) play an important role in connecting communities (Lienert, 2004) allowing species to overcome disturbances and habitat degradation (Cain, Milligan, & Strand, 2000). In this sense, plant meta‐communities can potentially form meta‐ecosystems at a large scale.

In the northern Hemisphere, small water bodies formed by delayed melting of ice blocks of retreating glaciers, commonly called kettle holes or potholes (Kalettka & Rudat, 2006; Kalettka, Rudat, & Quast, 2001; Tiner, 2003), are ideal for studying meta‐populations and meta‐communities as they often form a network of aquatic and wetland “island” habitats surrounded by an unsuitable matrix of intensively managed agricultural areas (Brose, 2001; de Meester et al., 2005). These wetland ecosystems with their gradient in soil humidity support a high diversity of flora (e.g., Patzig, Kalettka, Glemnitz, & Berger, 2012) and fauna (Céréghino et al., 2012; Gerke, Koszinski, Kalettka, & Sommer, 2010; Oertli et al., 2002). However, intensive agricultural management threatens kettle holes causing structural degradation, eutrophication, pollution by plant‐protection products, and direct habitat destruction (Altenfelder, Raabe, & Albrecht, 2014; Céréghino, Biggs, Oertli, & Declerck, 2008; Kalettka et al., 2001).

Given the high probability of disturbance and therefore potentially the highly dynamic nature of these small wetland ecosystems within the agricultural landscape, biotic connectivity patterns may strongly affect the species composition of the plant communities inhabiting these habitat islands (Bullock, Kenward, & Hails, 2002; Cain et al., 2000; Cottenie & De Meester, 2004). In addition, different abiotic factors, especially hydrological and geomorphological characteristics (Brinson, 1993; Kalettka & Rudat, 2006), may act as local filters (Schmid, Joshi, & Schläpfer, 2002) selecting for plant communities that may or not differ in plant diversity and functional traits in different types of kettle holes.

The aim of this study was to identify the main ecological processes driving plant diversity in meta‐communities of two types of kettle holes: steep permanent and therefore less plowed and less disturbed versus flat, ephemeral, plowable and more disturbed kettle holes, and their role as filters within an intensively managed agricultural matrix. To achieve this aim, we first compared plant diversity (alpha diversity) in relation to area of the pond (patch), and degree of isolation (number of ponds in the surroundings) to test whether larger areas harbor more species and whether more isolated patches harbor less species. Second, whether turnover of species and nestedness (beta diversity) differ in the two types of kettle holes for all plant species and including only wetland specialist species. Finally, we analyzed plant functional traits important for community dynamics including dispersal and movement abilities (pollen and seed dispersal) as well as colonization abilities (life span, seed longevity, and self‐compatibility systems) to test for niche differentiation processes (dissimilarities in traits) emphasizing on plant seed bank.

We hypothesized that the two types of kettle holes act as strong environmental filters shaping plant communities by different habitat conditions (Schmid et al., 2002). Under the paradigms of the meta‐community framework, we hypothesized that two main ecological processes occur: species sorting (SS) and mass effect (ME) (Leibold et al., 2004). Similarity in species composition in both types of kettle holes and non‐significant differences in dispersal abilities plus no effect of isolation would highlight the importance of SS, while a higher diversity in one of the type of kettle holes including all species of the other type might be an indication of source–sink mechanism related to a ME paradigm. A significant difference in trait distribution between communities would be an indication of niche differentiation between the two types of kettle holes.

## 2 METHODS

### 2.1 Study area

**Figure 1:**
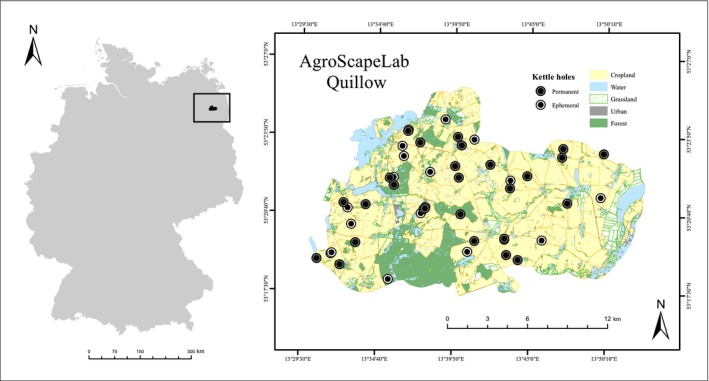
Study area: the Agricultural Landscape Laboratory “AgroScapeLab Quillow” (www.bbib.org/experimental-platform.html) in the Quillow catchment area located in North‐East Germany (Brandenburg). This agricultural landscape is characterized by a high density of kettle holes. Points denote our selected kettle holes (empty circles: flat/ephemeral, filled circles: steep/permanent). Percentage of land use in the area is 65% cropland, 17% forest, 9% grassland, 5% water, and 4% urban

Our study area was located in the “AgroScapeLab Quillow,” an agricultural landscape laboratory in the Quillow river catchment area, which was established by the Leibniz Centre for Agricultural Landscape Research (ZALF) e.V. approx. 100 km North of Berlin (Germany, Brandenburg). This area comprises around 290 km^2^ and contains a high density of small kettle holes (up to 2 per km^2^) (Kalettka, Berger, Pfeffer, & Rudat, 2005) connected by a shallow groundwater system (Kayler et al., 2017) and constantly influenced by seasonally changing hydrological conditions (Brose, 2001; Kalettka & Rudat, 2006; Figure 1). The water regime of the kettle holes from periodic to permanent in this region is influenced by a sub‐humid climate with precipitation of 450–600 mm/year and potential evapotranspiration of 600–650 mm/year (Kalettka & Rudat, 2006). The predominant land use of this area is intensive agriculture of maize, wheat, and rapeseed as the main crops.

### 2.2 Selection and classification of kettle holes

The study area was divided into smaller sections where small kettle holes were visible from Google Earth satellite images from 2002. With random simulated numbers, we selected some kettle holes in each section trying to equilibrate the number of the distinct types (permanent and non‐permanent). Some ephemeral kettle holes are very dynamic and can sporadically occur in the field in certain years depending on weather conditions (see below). If one of these dynamic kettle holes was not present during the initial sampling in the field, we selected another one close by if possible. We monitored plant‐species composition of the kettle holes in July and August 2011.

Based on hydro‐ and geomorphological characteristics, Kalettka and Rudat (2006) proposed a classification key for kettle holes in North‐East Germany. The first level of classification divides them into three groups: Storage Type, (S) Shore Overflow Type (SO), and Puddle Type (P). The storage and shore overflow types (S and SO) are deep with a permanent shore and mostly periodically to permanently flooded, while the puddle (or non‐permanent) type is flat without a permanent shore and mostly ephemerally flooded (Kalettka & Rudat, 2006). In dry years, the puddle types can disappear completely and can be easily plowed and used as arable land (Kalettka & Rudat, 2006). Based on these characteristics and the vulnerability to agricultural practices, we classified our 46 sampling kettle holes in two groups: (A) flat‐sloped, less permanent and plowed ones corresponding to Puddle types, and (B) steep‐sloped and more permanent ones including Storage and Shore Overflow types. For simplification, group (A) will be hereafter addressed as “flat ephemeral” and group (B) as “steep permanent” kettle holes.

### 2.3 Landscape parameters relevant for connectivity among wetland habitats

We calculated area and degree of isolation measured as the number of neighboring kettle holes within different radii: 20, 50, 100, 200, 500, 1,000, and 2,000 m using ArcGIS 10 (Esri, 2011) based on land use and habitat type maps provided by Leibniz Centre for Agricultural Landscape Research (ZALF).

### 2.4 Plant identification and plant functional traits

We recorded the presence or absence of all plant species occurring in the amphibian and terrestrial zone of the kettle hole. The amphibian zone is located between the open water body and (terrestrial) grassland vegetation next to the agricultural matrix (Patzig et al., 2012). We identified the species according to Rothmaler (2011) excluding those that were cultivated in the arable matrix (e.g., *Zea mays*, *Hordeum vulgare*, *Brassica napus*). Three taxa—*Rosa*, *Rubus*, *Taraxacum*—could be determined to genus level only. For each species, Ellenberg indicator values (Ellenberg, Weber, & Duell, 1991) were used to classify specialized wetland species (indicator value for moisture ≥7). The seed longevity index according to Bekker et al. (1998)—ranging from short‐lived seeds = 0 to long‐lived = 1—as well as data on species longevity was taken from the LEDA database (www.uni-oldenburg.de/en/landeco/research/leda/; Kleyer et al., 2008).

To test for functional differences in dispersal ability between plant communities occurring in permanent versus ephemeral kettle holes, we analyzed the breeding system (selfing possible vs. non‐selfers), the pollen vector (zoophily, anemophily, and selfing), the dispersal syndrome (zoochory, anemochory, hydrochory, hemerochory, and autochory), and life strategies for each plant species. The self‐compatibility, pollen vector, and life strategies dataset are based on BIOLFLOR (http://www2.ufz.de/biolflor/), the life span on the LEDA database (Kleyer et al., 2008), and the seed dispersal is mainly based on Rothmaler (2011) and completed with 3D Dispersal Diaspore Database (Hintze et al., 2013; www.seed-dispersal.info/terms-of-use.html) considering indices ranks >0.5. All of the previously mentioned traits are in relation to colonization and dispersal abilities. We counted the total number of species that possess a particular trait and we calculated the percentage of species. Species can belong to more than one group, for example, to more than one dispersal syndrome (zoo‐, anemo‐, hemerochory). Those species were counted separately and summed up in the corresponding groups (see Supporting Information Table S2 for details).

### 2.5 Plant seed bank

Soil samples were collected in April 2012 from 20 randomly chosen sites (ten permanent and ten ephemeral kettle holes; list in Supporting Information Table S3). Soil samples were collected within the outer circumference of the kettle holes within the amphibian transition zone between open water body and grassland vegetation (ten random samples per site, 10 cm deep, with a diameter of 3 cm) using a clean soil corer. Soil samples were stored in a cool dry place for three weeks until used for seed bank assessment and soil pH analysis. During three months, the number and identity of emerging seedlings of the soil seed bank was weekly assessed in trays at the common garden site of the University of Potsdam using the seedling emergence method described in Kurtz and Heinken (2011).

For the seed bank assays, seeds were divided into two wet treatments: flooded and non‐flooded types to replicate natural conditions of permanent and ephemeral kettle holes. We tested whether germination varied according to treatment (flood, non‐flood) and type of kettle holes (permanent, ephemeral). We measured the actual pH (soil/0.01 M calcium chloride solution ratio: 1:2.5) of the soil samples (using a WTW pH meter 325, Germany) to test whether putative differences in functional community composition are related to soil pH (see Ma, Baskin, Yu, Ma, & Du, 2017).

### 2.6 Statistical analysis

We used GLMs (Generalized Linear Models) to test whether the two different types of kettle holes differed in plant‐species richness in relation to area and isolation degree. Due to overdispersion in the data, we explored two classes of models based on quasipoisson and negative binomial distribution. Since both models yielded similar results, we selected the quasipoisson model (Hoef & Boveng, 2007) using the glm function in R. We tested if species richness of all plants or of specialized wetland plants only depends on kettle hole area and if this effect differs between kettle hole types and whether the number of kettle holes in the surrounding has also an influence (isolation degree). For this, we previously tested which buffers (20, 50, 100, 200, 500, 1,000, and 2,000 m radii) influence plant‐species richness and selected the minimum significant to fit the model. Due to very low number of ponds in small radii, we discarded the first three buffers (20, 50, 100 m). A similar procedure was performed for the seed bank experiment, to test the influence of two factors: type of kettle hole and treatment (flooded or not) on germination.

To test the hypothesis that species composition varies between flat and steep kettle holes, first, we calculated overall beta diversity and its components: turnover and nestedness based on Jaccard dissimilarity matrices for presence–absence dataset with the function “beta.multi” and three matrices containing the pairwise between‐site values of each component of beta diversity with the function “beta.pair” from the package betapart (Baselga & Orme, 2012). Then, we compared beta diversity between groups (types of kettle holes) using the function “betadisper” based on permutation tests (PERMANOVA) under 95% confidence intervals around treatment centroids. Additionally, an overall beta diversity was calculated based on Ochiai index of similarity (Ochiai, 1957). This index excludes double absences; it allows for chord or Hellinger transformation (Borcard, Gillet, & Legendre, 2008) and proofed to be useful for plant communities (De Caceres, Font, & Oliva, 2008). We computed an Ochiai index followed by a Hellinger transformation for our species presence–absence data. An ordination of Principal Coordinates Analysis (PCoA) was performed based on these Ochiai distances to visualize the plant communities.

Finally, to test whether percentage of plant species with a particular functional trait related to dispersal, reproduction, or recruitment differ according type of kettle holes (permanent vs. ephemeral), we applied ANOVA tests because the data presented normality and homogeneity of variances (Supporting Information Table S5).

## 3 RESULTS

**Figure 2:**
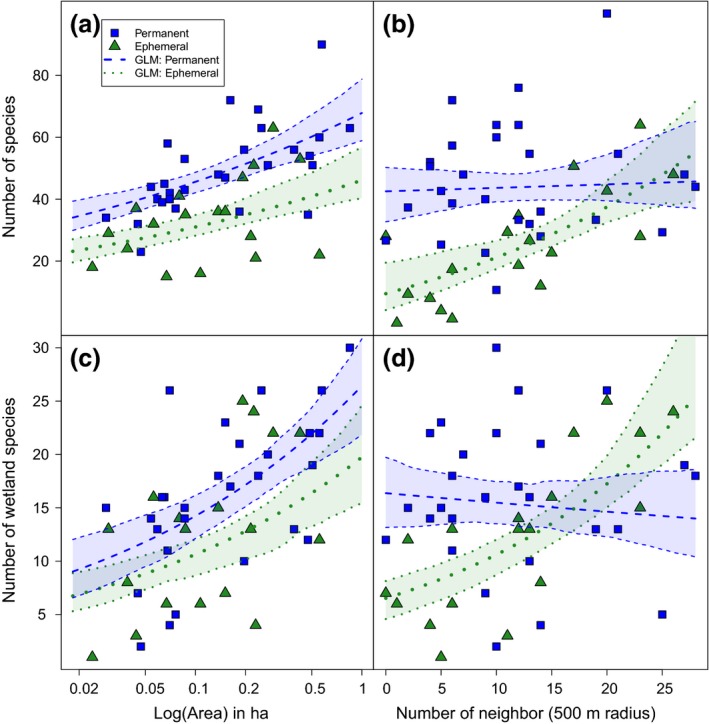
(a) Relationship between plant‐species richness and area (in ha) within the two types of kettle holes: ephemeral (flat) and permanent (steep); (b) number of neighboring ponds within a 500 m radius. There was a positive correlation between number of plant species and area in both types of kettle holes (all *p* < 0.001). In contrast, only species occurring within ephemeral ponds were positively influenced by the number of neighboring kettle holes within a 500 m radius (**#** neighbors × type of kettle hole *p* < 0.001). The same pattern was found when only wetland species were analyzed (c: all *p* < 0.001; d: **#** neighbors × type of kettle hole *p* < 0.05**)**

In total, 254 vascular plant species were identified in the 46 kettle holes studied (details in Supporting Information Tables S1 and S2). Plant‐species richness differed between the two kettle hole types with a 41.5% lower species diversity in flat ephemeral kettle holes compared with steep, more permanent ones (138 vs. 236 species, respectively; *F*_1,44_ = 13.96, *p* < 0.001). Of these, 120 plant species occurred in both habitat types, 116 exclusively in steep ones, and 18 plant species exclusively in flat kettle holes (Table 1). In both habitat types, species richness increased with kettle hole area (Figure 2a). Increasing area was especially positively related to plant‐species richness in permanent kettle holes when only specialized wetland plants were considered (Figure 2c). In contrast to the steep permanent sites, however, the total plant‐species richness as well as the number of wetland species was positively influenced by the number of neighboring ponds within a 500 m radius only in the flat ephemeral kettle holes (Table 2, Figure 2b,d).

**Table 1:** Summary table of size (area), degree of isolation (number of neighbors within a 500 m radius), and total number of plant species found in the entire community and only the specialized wetland plants in both types of kettle holes: ephemeral and permanent

		Permanent	Ephemeral	Overall
Area [m^2^]	Mean ± *SD*	2,228 ± 2,127	1,637 ± 1,442	1,997 ± 1,893
	Min	290	240	240
	Max	8,500	5,600	8,500
# Neighboring kettle holes	Mean ± *SD*	11.5 ± 7.4	11.7 ± 8.0	11.5 ± 7.5
	Min	0	0	0
	Max	28	26	28
Total species richness	Mean ± *SD*	49.3 ± 14.2	33.5 ± 13.6	43.2 ± 15.8
	Total	116	18	254
	Both	—	—	120
Wetland species richness	Mean ± *SD*	16.2 ± 7.0	12.4 ± 7.4	14.7 ± 7.3
	Total	28	6	80
	Both	—	—	46

**Table 2:** Summary of statistical models used for landscape connectivity parameters (area and isolation) and for the seed bank experiment in a subset of 20 kettle holes

Best model	Response variable	Predictors	Coefficient value	Statistic value	*p*‐Value	qAIC
Seed bank experiment (*n* = 20)						
m01	Germination	Intercept	3.17	*t* = 20.62	<0.001***	222.35
		Permanent	−0.63	*t* = −2.43	<0.05*	
Landscape connectivity (*n* = 46)						
m12 (all species)	Species number	Intercept	3.44	*t* = 6.28	<0.001***	167.47
		Log area [m^2^]	0.17	*t* = −0.88	<0.001***	
		Neighbors 500 m (a)	0.03	*t* = 0.85	<0.001***	
		Permanent (b)	0.75	*t* = −0.24	<0.01**	
		a:b	−0.03	*t* = −0.24	<0.001***	
sp12 (wetland species)	Species number	Intercept	2.42	*t* = 9.94	<0.001***	152.94
		Log area [m^2^]	0.26	*t* = 4.43	<0.001***	
		Neighbors 500 m (a)	0.04	*t* = 4.12	<0.001***	
		Permanent (b)	0.91	*t* = 3.89	<0.001***	
		a:b	−0.05	*t* = −3.67	<0.001***	

Note

Model selection was performed to explain the effect of size (area) and isolation degree (number of neighbors) on plant richness in both types of kettle holes in the entire community and for wetland species only, as well as the effect of types of kettle holes and wet treatment in germination from the seed bank. Due to overdispersion, Generalized Linear Models (GLM) with a “quasipoisson” distribution were applied and model selection based on qAIC (lowest value) was performed (for details see Supporting Information Table S4). Significance levels are indicated with asterisks: ***p < 0.001, **p < 0.01, *p < 0.05.

The best model explaining the relationship between species number (all and wetland species only) and area and number of neighboring kettle holes within a 500 m radius was species number ~logArea (ha) + Number of neighbors x* Kettle hole type (qAIC of 167.47 and 152.94, respectively; Table 2) (all models in Supporting Information Table S4).

Soil pH in the seed bank experiment showed a marginal but not significant difference between flat ephemeral versus steep permanent kettle holes (7.1 ± 0.24 vs. 6.8 ± 0.44; *F*_1,419_ = 3.71 *p* = 0.069; Supporting Information Figure S1). From a total of 34 different species that germinated, 19 species plus *Brassica napus *(Rapeseed of the surrounding matrix) could be identified to species level; no woody species were found (Species list in Supporting Information Table S3). A total of 9,981 seedlings germinated and seed abundance significantly varied between types of kettle holes (*F*_1,542_ = 5.48; *p* = 0.01) with a higher seedling abundance in flat ephemeral than permanent kettle holes (22.3 ± 29.2 vs. 11.6 ± 15.3, respectively). Wet treatment (flooded vs. non‐flooded) had no effect in seedling abundance (*F*_1,542_ = 1.14; *p* = 0.29). The best fitted model was Germination ~Kettle hole type (qAIC = 222.35; Table 2).

**Figure 3:**
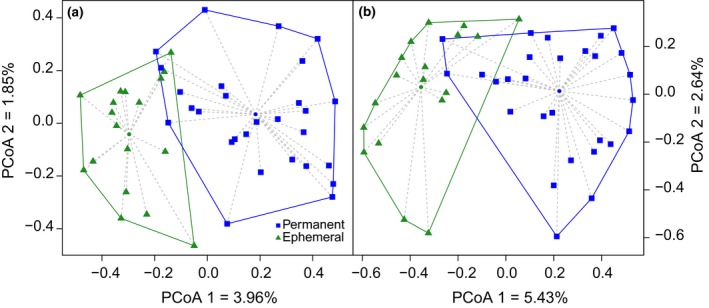
Principal Coordinate Analysis using species composition of all (a) or specialized wetland plant species only (b). An Ochiai matrix was generated as a standardization of data, following De Caceres et al. (2008), and afterward, a Hellinger transformation was applied. Results of PERMANOVA based on 99,999 permutations showed a difference in plant‐species composition according to the kettle hole types for all species (*F*_1,44_ = 4.37; *p* = 0.04), and a tendency for difference when considering wetland species only (*F*_1,44_ = 3.42; *p* = 0.07)

High levels of beta diversity across study sites were found both in the entire community and for specialized wetland species (0.969 and 0.971, respectively) where species turnover (0.955 and 0.951) contributed considerably more to dissimilarity than nestedness (0.014 and 0.020; Table 3) in both communities. A Permutation Multivariate Analysis of Variances (PERMANOVA) showed a significant difference between the types of kettle holes for turnover of species and nestedness for the entire community (Turnover: *F*_1,44_ = 7.38; *p* < 0.01; Nestedness: *F*_1,44_ = 10.19; *p* < 0.01) and wetland community (Turnover: *F*_1,44_ = 11.44; *p* < 0.01; Nestedness: *F*_1,44_ = 12.82; *p* < 0.001). Overall, beta diversity based on Jaccard similarity showed no difference between the types of kettle holes neither for the entire community, nor for the specialized wetland species (*F*_1,44_ = 2.11; *p* = 0.15; *F*_1,44_ = 1.15; *p* = 0.29). However, overall beta diversity based on Ochiai distances after a Hellinger transformation showed a separation in species composition between the two types of kettle holes (Figure 3a,b) when all plants species were considered (*F*_1,44_ = 4.37; *p* = 0.04) and a tendency for separation when only wetland species were considered (*F*_1,44_ = 3.42; *p* = 0.07) (Table 3).

**Table 3:** Species turnover, nestedness, and overall beta diversity based on site dissimilarity (Jaccard dissimilarity) between the two types of kettle holes for the entire community and for the specialized wetland plants

	Turnover (Jaccard)	Nestedness (Jaccard)	Overall β‐diversity	
			Jaccard distance	Ochiai distance
All species	0.955 (*p* < 0.01**)	0.014 (*p* < 0.01**)	0.969 (*p* = 0.15)	*p* = 0.04*
Ephemeral	0.872	0.051	0.923	
Permanent	0.933	0.017	0.951	
Wetland species	0.951 (*p* < 0.01**)	0.020 (*p* < 0.001***)	0.971 (*p* = 0.29)	*p* = 0.071
Ephemeral	0.837	0.089	0.927	
Permanent	0.924	0.028	0.952	

Note

Results of a PERMANOVA (95% CI) show the comparison of the distance to centroids calculated according to the type of kettle hole (permanent vs. ephemeral) for overall beta diversity and its components (turnover and nestedness) based on Jaccard dissimilarity. Overall, beta diversity was also calculated based on Ochiai distances, which allowed for a Hellinger transformation for presence–absence data. Significance levels are indicated with asterisks: ***p < 0.001, **p < 0.01, *p < 0.05.

**Figure 4:**
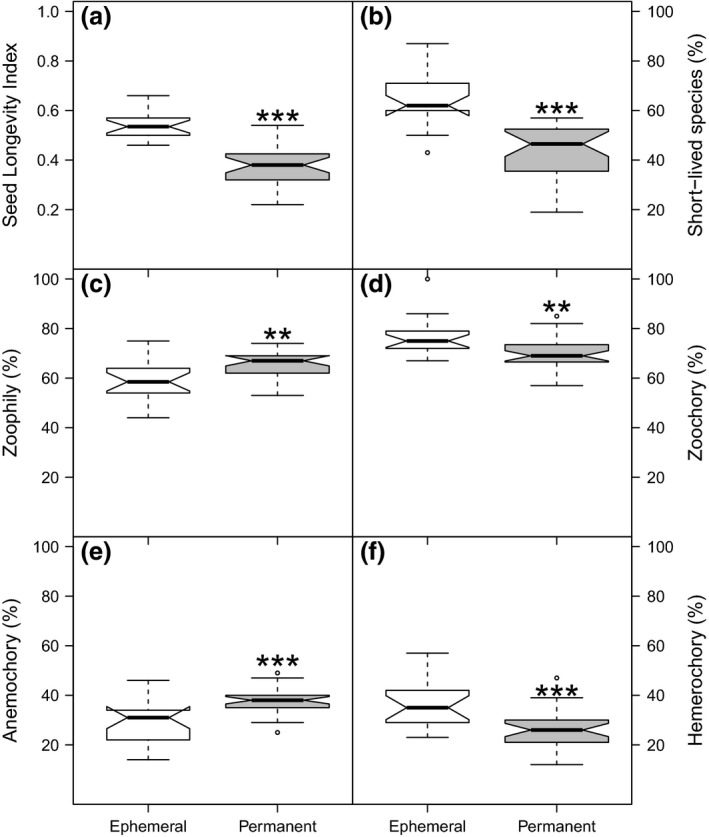
Plant traits important for colonization: seed longevity (a) and individual life span (b). The seed bank longevity index (ranging from short‐lived = 0 to long‐lived seeds = 1) was significantly higher in the ephemeral kettle holes (*p* < 0.001) harboring more persistent seeds. In contrast, in these ephemeral, flat and more disturbed kettle holes, more short‐lived plants (non‐perennials) with a faster life cycle (annuals, biannual) were found (*p* < 0.001). In addition, plant traits for pollen movement (c) and seed dispersal (d–f) differed among types of kettle holes (all *p* < 0.01). Permanent kettle holes harbored a higher percentage of species pollinated by animals and seeds dispersed by wind (all *p* < 0.01); and ephemeral kettle holes contained more species with seeds dispersed by animals and human‐related vectors (all *p* < 0.01)

Separation in plant‐community composition between both types of kettle holes was reflected in the distribution of functional traits (Table 4). The majority of the species occurring in the ephemeral kettle holes had faster life cycles (higher percentage of annual and biennial plants; 64% ± 0.4% vs. 44% ± 0.5; *F*_1,32_ = 46.96; *p* < 0.0001; Figure 4b), and their seed bank was more persistent (0.5 ± 0.2 vs. 0.3 ± 0.2 ranging from short‐lived = 0 to long‐lived seeds = 1; *F*_1,40_ = 91.31; *p* < 0.0001; Figure 4a). In addition, seed‐dispersal abilities of the species varied according to the types of kettle holes with a slightly but significantly higher percentage of plants with zoochorous seed dispersal in ephemeral kettle holes than in permanent ones (76% ± 0.5% vs. 70% ± 0.4; *F*_1,38_ = 10.79; *p* < 0.01). In contrast, fewer plant species relied on wind dispersal of seeds in ephemeral compared with permanent kettle holes (29% ± 0.4% vs. 38% ± 0.5; *F*_1,38_ = 10.79; *p* < 0.001; Figure 4d,e). The number of species that can produce seeds via selfing did not differ between the two types of kettle holes (all *p* > 0.1), but there was a slightly higher number of self‐incompatible species (obligate outbreeders) in permanent kettle holes (28% ± 0.4% vs. 21% ± 0.4; *F*_1,43_ = 0.26; *p* < 0.0001). Moreover, in ephemeral kettle holes, we found a higher percentage of species that are mainly dispersed by humans (hemerochory) than in permanent ones (36% ± 0.4% vs. 26% ± 0.4; *F*_1,42_ = 0.26; *p* < 0.0001; Figure 4f). Finally, with respect to pollen vectors, there was a relatively lower percentage of insect‐pollinated species in ephemeral kettle holes compared with permanent ones (59% ± 0.4% vs. 65% ± 0.4; *F*_1,38_ = 10.54; *p* < 0.01; Figure 4c).

**Table 4:** Comparison of plant traits affecting colonization and dispersal abilities within the two different types of kettle holes: flat ephemeral and steep more permanent

Plant functional traits		Ephemeral		Permanent		ANOVA		
		% sp	*SD*	% sp	*SD*	*F*	*df*	*p*
*Colonization abilities*								
Self‐compatibility	Self‐compatible	80.7	0.23	80.7	0.31	0.00	1,43	0.995
	Self‐incompatible	21.3	0.42	28.6	0.46	0.26	1,43	<0.001***
Recruitment	SLI^a^	0.54^a^	0.27	0.37^a^	0.29	91.31	1,40	<0.001***
Life span	Short‐lived	63.7	0.43	43.8	0.49	46.96	1,32	<0.001***
	Long‐lived	46.3	0.50	67.8	0.42	61.33	1,38	<0.001***
*Dispersal abilities*								
Pollen dispersal	Zoophily	59.4	0.48	65.2	0.46	10.54	1,38	0.002**
	Anemophily	37.0	0.49	36.4	0.48	0.07	1,38	0.7
	Hydrophily	1.3	0.06	4.1	0.17	11.58	1,38	0.002**
	Selfing	56.9	0.49	55.4	0.49	1.69	1,38	0.2
Seed dispersal	Zoochory	76.3	0.41	69.7	0.45	10.79	1,38	0.002**
	Anemochory	28.9	0.45	37.6	0.48	23.21	1,38	<0.001***
	Hydrochory	45.6	0.49	46.3	0.49	0.07	1,38	0.8
	Hemerochory	36.3	0.48	26.3	0.43	16.58	1,42	<0.001***
	Autochory	10.3	0.27	17.8	0.38	20.70	1,38	<0.001***

Note

Data show percentage of species (% sp) plus Standard Deviation (*SD*). Note that the sum of species of both types exceeds 100% as often one species possesses more than one trait (see Methods). Analysis of Variance (ANOVA) was performed to evaluate whether the different functional traits differed according to type of kettle hole. Significance levels are in bold and indicated with asterisks: ***p < 0.001, **p < 0.01, *p < 0.05.

a SLI = Seed Longevity Index, data shown in mean.

## 4 DISCUSSION

The aim of this study was to identify the main ecological processes driving plant diversity in two types of kettle holes—steep permanent versus flat ephemeral—within an intensively used agricultural landscape under a meta‐community framework. We compared plant features such as life span, seed dispersal ability, pollen transfer, seed bank, and seed longevity in these two wetland types. Our results suggest that the type of kettle holes acts as a strong environmental filter for plant communities, but this system cannot be explained by one meta‐community paradigm only. Whereas flat ephemeral kettle holes can be regarded as meta‐communities that strongly depend on seed dispersal and recruitment from a seed bank, the plant‐species richness of neighboring permanent kettle holes was not influenced by degree of isolation and had a more stable local species diversity. Furthermore, the significant difference in trait distribution between communities is an indication of niche differentiation between the two types of kettle holes. Hence, plant functional traits offer good insights in understanding the role of local environmental conditions (local filters) and regional species sorting in these freshwater islands within an intensively managed agricultural matrix.

### 4.1 Species sorting and mass effect processes at different scales

Apart from the rare and endangered 21 plant species present in the state red list of Brandenburg (Ristow et al., 2006), the overall considerable diversity of 254 plant species found in 46 small kettle holes within the matrix of intensively managed agricultural fields, substantially enhances biodiversity at the landscape scale. As expected, in both types of kettle holes, we found a positive correlation between species richness and habitat size where a larger area harbors a higher number of species, as it was previously well documented for small wetland habitats (e.g., Jeffries, 2012 and references therein). This can be generally explained by the framework of “environmental heterogeneity” where a wider range of habitats is suitable for more different plant species in larger habitats (Stein, Gerstner, & Kreft, 2014). However, this relationship can vary among taxa (Oertli et al., 2002) and diversity is not always reflected by species richness but by the diversity of functional traits. For example, a previous study in the same region by Patzig et al. (2012) found no clear pattern regarding macrophyte species richness.

Differences in alpha and beta diversity between types of kettle holes without a change between the entire community and the wetland plant community suggest that different environmental conditions act as local filters (Schmid et al., 2002) driving functional niche occupancy (Li et al.., 2017) reflected in different plant functional traits (Figures 3 and 4). Under the framework of meta‐community paradigms when habitat patches are environmentally heterogeneous, species sorting (SS) or mass effect (ME) processes may occur (Leibold et al., 2004). Different environmental conditions of the kettle holes provide a different habitat quality that in combination with different dispersal strategies affect community composition supporting the species‐sorting process (Leibold et al., 2004) at a regional level. In concordance, our beta diversity results show that plant communities between kettle holes are mainly explained by species turnover (species replacement from one pond to another) without differences between the entire and the wetland community (Table 3). Similar results were previously reported for meta‐communities of aquatic plants and macroinvertebrates (Hill, Heino, Thornhill, Ryves, & Wood, 2017; Viana et al., 2016), supporting the species‐sorting process at a regional scale.

A low number of unique species in ephemeral kettle holes (18 out of 254 species) but not in permanent kettle holes (116/254) suggests a mass effect process, where permanent kettle holes might be acting as a source and ephemeral ones as sink supported by the high number of shared seedlings that germinated in both types of ponds (21/34; Table 1). A negative relationship with distance to neighboring ponds in flat kettle holes (Figure 2d) suggests that spatial colonization (dispersal filtering) is also an important process driving community assembly in these ephemeral habitats. In addition, turnover of species and nestedness differed depending on type of kettle holes with a higher turnover in permanent and a higher nestedness in ephemeral kettle holes (Table 3). These results show that the larger permanent ponds also follow the SS paradigm harboring species with a higher replacement than ephemeral ones. The higher nestedness in ephemeral ponds suggests that they are a subset of the species assemblage of the permanent ponds supporting the mass effect process at a local scale.

Finally, if we only consider ephemeral kettle holes and assume that patches among them are similar, the dynamic state of these kettle holes (drying and reappearing) might reflect a patch dynamic paradigm where patches can be occupied or unoccupied where local diversity is limited by dispersal (Leibold et al., 2004). It is known that temporal variation in patch suitability and availability in combination with spatial colonization and founder effects play an important role shaping communities (Jeffries, 2008; Mahaut, Fried, & Gaba, 2018). In our system, ephemeral kettle holes possessed a more persistent seed bank source of propagules (Figure 4a) in combination with short‐lived species (Figure 4b) suggesting that the species' life cycles are more in synchrony with patch availability enabling persistence on the sites over periods when the ephemeral kettle holes are not present (e.g., Alderton, Sayer, Davies, Lambert, & Axmacher, 2017; Poschlod & Rosbakh, 2018). Even though we found a low number of competitive species in both ephemeral and permanent ponds (~20% and ~30%) (data not shown), the relationship between migration (dispersal) and local dominance and colonization–competition trade‐offs are fundamental to assess patch dynamics (Logue et al., 2011). Since our data (presence–absence) lack abundance information, further experiments are needed to confirm these hypotheses.

### 4.2 Linking species sorting with movement ecology

In plants, it is mainly seed dispersal that defines movement ecology (Nathan et al., 2008), and therefore, the most important factors influencing seed movement are dispersal vectors (biotic and abiotic) in combination with motion abilities, followed by environmental filters (Damschen et al., 2008). Both, environmental conditions and spatial distribution of suitable habitats can lead to environmental and dispersal filtering (seed arrival, recolonization events) and both are shaping local species communities (Fraaije et al., 2015). Additionally, it has been shown that pollen transfer is as an important limiting factor connecting populations with consequences in biodiversity and regeneration (Schermer et al., 2018) or economic loss in agricultural landscapes related to invasive weeds (e.g., Fénart, Austerlitz, Cuguen, & Arnaud, 2007). Our results showed a higher number of zoophilous plant species (insects as pollen vectors) in permanent kettle holes. These results suggest that permanent kettle holes provide habitat and food source to harbor a higher number of pollinators (e.g., wild bees and bumblebees), whose community might be related to higher plant diversity and habitat heterogeneity found in the permanent kettle holes compared with the ephemeral ones. This might be related to the higher number of obligate outbreeders (self‐incompatible) species found in these permanent kettle holes (Supporting Information Figure S2).

Our results showed a difference in dispersal syndrome depending on environment where biotic dispersal vectors (zoo‐, hemerochory) seemed to be more effective in ephemeral kettle holes and abiotic vectors (anemochory) in permanent kettle holes (Figure 4d–f). A possible explanation to these results might be that kettle holes offer a different accessibility for seed dispersers, mainly biotic, and a different degree of exposure and vulnerability to intensive land use. Even though both types of kettle holes constitute a source of food and water for animals (deer, wild boars, migratory birds), which might disperse the seeds while foraging (e.g., Dovrat, Perevolotsky, & Ne'eman, 2012; Figuerola, Green, & Santamaría, 2003; Flaherty, Rentch, & Anderson, 2018; Soons et al., 2016), permanent kettle holes harbor a significantly higher number of long‐lived (Supporting Information Figure S2) and tall plant species that might offer a better shelter for animals, or form less accessible dense thickets compared with ephemeral kettle holes. Consequently, ephemeral kettle holes are in more direct contact with the intensive land‐use surroundings and farming activities (e.g., tractors for harvest), which could easily act as potential—hemerochorous—seed dispersers (Figure 4f).

### 4.3 Ephemeral kettle holes as stepping stones to conserve plant diversity

The dynamic state of ephemeral kettle holes provides different environmental conditions for colonization events and different dispersal vectors (highly mobile birds or humans via agricultural machinery) compared with permanent kettle hole ecosystems consisting of more long‐lived plants. Both types of ponds form a dense network of freshwater island habitats where ephemeral ponds might act as stepping stones due to the common, unique, and high turnover of species enhancing the overall plant diversity at the landscape scale. The importance of ephemeral kettle hole density for the maintenance of plant‐species richness is supported by a low weed diversity recently found in agricultural fields within the same area (Müller‐Nilsson, 2018) suggesting a low permeability for wild plants of the agricultural matrix surrounding the ephemeral kettle holes.

A previous study in the region suggested that management and conservation policies should consider the types of kettle holes (Patzig et al., 2012). Other studies focused on temporary flooded depressions provide measures to conserve plant communities based on management of water‐level fluctuations and land‐use practices (Altenfelder, Kollmann, & Albrecht, 2016; Altenfelder, Schmitz, Poschlod, Kollmann, & Albrecht, 2016). We highlight the importance of flat ephemeral kettle holes as key habitats acting as stepping stones to preserve plant diversity within this agricultural landscape (Hallmann et al., 2017). Despite their biodiversity and the ecosystem services these small water bodies provide, conservation policies are not well established yet, excluding them from freshwater science and international nature conservation policies (Biggs, von Fumetti, & Kelly‐Quinn, 2017). To overcome this problem, Hill et al. (2018) recently proposed practical steps to focus on “pondscapes” and their impact on society. Our study contributes to a better understanding of these ponds but long‐term studies to understand the dynamics of these meta‐communities are needed (Ruhí, Datry, & Sabo, 2017) for a future integration of these pondscapes into policies and a sustainable management of these agricultural landscapes.

## 5 CONCLUSION

Our study shows that differences in alpha and beta diversity between steep permanent compared with ephemeral flat kettle holes are mainly explained by species sorting and niche processes at regional scale, while mass effect and dispersal limitation processes are detectable at local scale in ephemeral kettle holes only. We highlight the importance of supporting a high density of flat ephemeral kettle holes within intensively managed agricultural landscapes to sustain population dynamics and plant diversity. Flat ephemeral kettle holes are more vulnerable to environmental filtering particularly related to human activities compared with steep permanent kettle holes. We suggest to establish management and conservation policies focusing on these freshwater bodies considering their function as stepping stones enhancing plant diversity in intensively used agroecosystems.

## CONFLICT OF INTEREST

None declared.

## AUTHOR'S CONTRIBUTION

JJ, SS, BS, TK, JE, and TH designed the study. SS and KPW collected the data in the field. SS, SLG, and BS analyzed the data, SLG, JJ, KPW, TK, TH, BS, and JE wrote the paper, contributed critically to the drafts, and gave final approval for publication.

## Data Availability

Data are available in Supporting Information and raw data are deposited in the ZALF Repository http://www.doi.org/10.4228/ZALF.DK.102.
