# Supplementary figures and images for: Environmental filtering predicts plant‐community trait distribution and diversity: Kettle holes as models of meta‐community systems
Source: Ecol Evol. 2019 Jan 21;9(4):1898–910. doi: 10.1002/ece3.4883 (PMC6392361; doi:10.1002/ece3.4883)

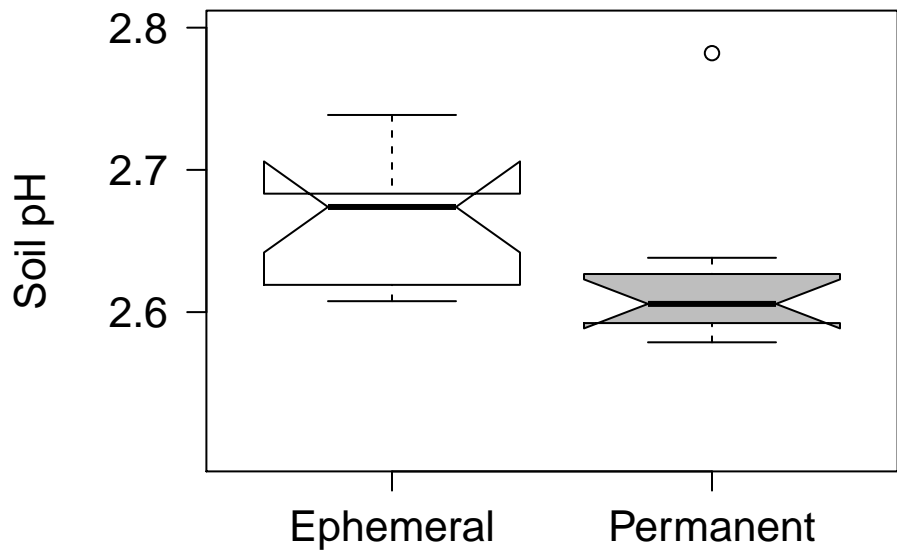

Supplement: Supplementary file 1 [file ECE3-9-1898-s001.pdf]

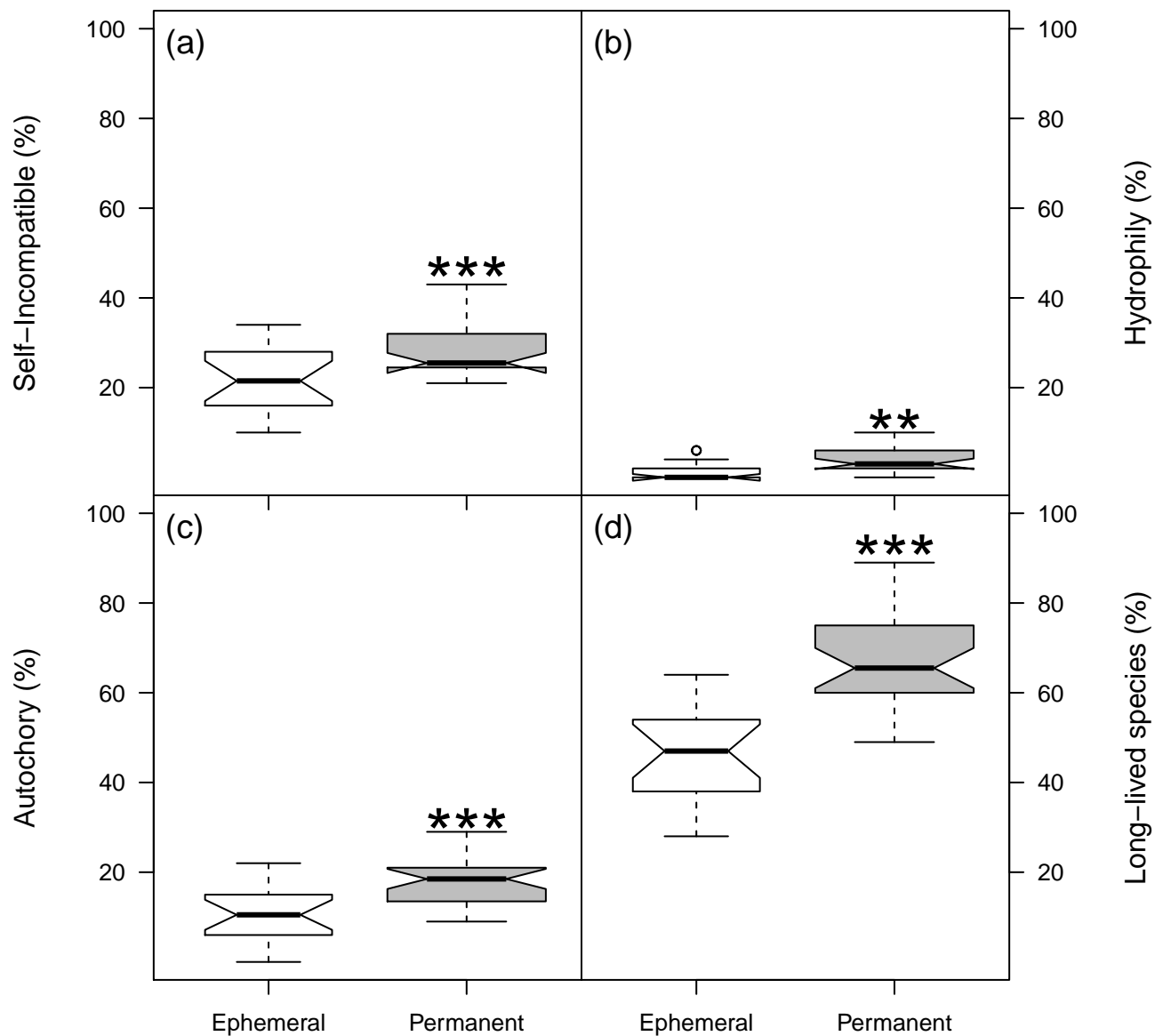

Supplement: Supplementary file 2 [file ECE3-9-1898-s002.pdf]
