# Supplementary material for: Environmental filtering predicts plant‐community trait distribution and diversity: Kettle holes as models of meta‐community systems
Source: Ecol Evol. 2019 Jan 21;9(4):1898–910. doi: 10.1002/ece3.4883 (PMC6392361; doi:10.1002/ece3.4883)
Supplement: Supplementary file 3 [file ECE3-9-1898-s003.docx]

**Environmental filtering predicts plant-community trait distribution and diversity: Kettle holes as models of meta-community systems**

**Supplementary material**

Lozada-Gobilard *et al.*

**SUPPLEMENTARY TABLES**

**Table S1:** Characteristics of the kettle holes:

**Table S2:** Characteristics of the plant species including colonization and dispersal traits.

**Table S3:** List of species found in the seedbank in a subset of 20 kettle holes.

**Table S4:** Detailed Generalized Linear Models with a quasi-poisson distribution selection based on Explanatory Deviance and Qaic.

**Table S5:** Best fitted Linear Models and ANOVAs of colonization and dispersal traits.

**SUPPLEMENTARY FIGURES**

**Fig. S1:** Soil analysis of a subset of 20 kettle holes.

**Fig. S2:** Additional colonization and dispersal plant traits.

**Table S1:** Characteristics of the kettle holes including geographical location, type, area, number of of neighbouring kettle holes within a radius of 500 m (using ArcGIS 10; ESRI 2011), surrounding crop, presence of trees, total number of plant species.

| **ID** | **Type** | **Area [m^2^]** | **# Neighbours** | **Longitude_X** | **Latitude_Y** | **Crop** | **Trees** | **# species** |
| --- | --- | --- | --- | --- | --- | --- | --- | --- |
| S010 | Steep permanent | 1620 | 3 | 3402490.822 | 5907660.721 | cereal | Yes | 72 |
| S011 | Steep permanent | 860 | 8 | 3419582.472 | 5912196.614 | rape | Yes | 43 |
| S012 | Flat ephemeral | 5560 | 2 | 3415332.817 | 5913951.378 | cereal | No | 22 |
| S013 | Steep permanent | 1960 | 12 | 3406282.85 | 5914152.231 | others | Yes | 56 |
| S014 | Steep permanent | 700 | 13 | 3406584.915 | 5913623.503 | others | Yes | 42 |
| S015 | Flat ephemeral | 2240 | 22 | 3408589.986 | 5911508.778 | cereal | No | 51 |
| S016 | Steep permanent | 1370 | 20 | 3408804.441 | 5911785.319 | cereal | Yes | 48 |
| S017a | Steep permanent | 5050 | 18 | 3408898.742 | 5911857.642 | cereal | No | 51 |
| S017b | Flat ephemeral | 1370 | 15 | 3408939.565 | 5911942.263 | cereal | No | 36 |
| S018 | Flat ephemeral | 2150 | 5 | 3417668.16 | 5909431.025 | cereal | No | 28 |
| S019 | Steep permanent | 2720 | 8 | 3400763.977 | 5908121.123 | rape | No | 51 |
| S020 | Flat ephemeral | 870 | 16 | 3401822.475 | 5908467.474 | maize | No | 35 |
| S021 | Flat ephemeral | 800 | 14 | 3401887.519 | 5908559.639 | cereal | No | 41 |
| S022 | Steep permanent | 650 | 6 | 3415858.193 | 5907966.028 | cereal | No | 45 |
| S023 | Steep permanent | 2380 | 11 | 3414978.263 | 5908333.186 | cereal | No | 69 |
| S024 | Steep permanent | 1510 | 5 | 3414855.188 | 5909499.901 | cereal | Yes | 47 |
| S025 | Steep permanent | 290 | 4 | 3414854.506 | 5909574.017 | cereal | No | 34 |
| S026 | Steep permanent | 1840 | 8 | 3419206.818 | 5915652.218 | cereal | No | 36 |
| S027 | Steep permanent | 5560 | 7 | 3419288.853 | 5916308.599 | cereal | Yes | 60 |
| S028 | Steep permanent | 4740 | 3 | 3422334.224 | 5915910.9 | cereal | Yes | 35 |
| S029 | Steep permanent | 540 | 6 | 3412607.454 | 5909410.554 | cereal | No | 44 |
| S030 | Steep permanent | 4860 | 4 | 3415291.084 | 5913345.132 | cereal | Yes | 54 |
| S031 | Flat ephemeral | 1070 | 4 | 3412082.507 | 5908609.363 | cereal | No | 16 |
| S032 | Steep permanent | 470 | 2 | 3411578.082 | 5911426.03 | maize | No | 23 |
| S033 | Steep permanent | 700 | 10 | 3416594.18 | 5914275.245 | maize | Yes | 40 |
| S034 | Steep permanent | 860 | 1 | 3411453.948 | 5914155.465 | cereal | No | 53 |
| S035 | Steep permanent | 590 | 14 | 3411166.77 | 5915019.121 | cereal | No | 40 |
| S036 | Flat ephemeral | 4250 | 9 | 3410453.343 | 5918540.624 | cereal | No | 53 |
| S037 | Steep permanent | 3910 | 10 | 3411674.342 | 5916589.685 | rape | Yes | 56 |
| S038 | Flat ephemeral | 2930 | 18 | 3407666.493 | 5917754.869 | rape | No | 63 |
| S039 | Steep permanent | 760 | 18 | 3407658.364 | 5917669.167 | rape | Yes | 37 |
| S040 | Flat ephemeral | 300 | 10 | 3407224.132 | 5916554.938 | maize | No | 29 |
| S041 | Steep permanent | 2490 | 8 | 3408551.963 | 5916811.128 | others | No | 63 |
| S042 | Flat ephemeral | 560 | 8 | 3409296.994 | 5914596.012 | cereal | No | 32 |
| S043 | Steep permanent | 680 | 3 | 3404454.433 | 5912173.028 | maize | Yes | 58 |
| S044 | Flat ephemeral | 670 | 0 | 3422094.256 | 5912619.964 | maize | No | 15 |
| S045 | Steep permanent | 450 | 9 | 3402797.079 | 5912318.703 | cereal | Yes | 32 |
| S046 | Flat ephemeral | 2300 | 12 | 3403107.908 | 5911923.833 | maize | No | 21 |
| S047 | Flat ephemeral | 240 | 7 | 3403368.429 | 5910698.084 | cereal | No | 18 |
| S048 | Steep permanent | 630 | 3 | 3403681.123 | 5909313.416 | others | No | 39 |
| S049 | Flat ephemeral | 1510 | 13 | 3406103.879 | 5905435.088 | cereal | No | 36 |
| S050 | Flat ephemeral | 390 | 11 | 3412616.188 | 5917021.627 | cereal | No | 24 |
| S051 | Steep permanent | 5740 | 15 | 3411401.437 | 5917211.765 | cereal | Yes | 90 |
| S052 | Flat ephemeral | 1920 | 17 | 3407323.457 | 5915780.126 | maize | No | 47 |
| S053 | Flat ephemeral | 440 | 11 | 3406574.478 | 5914209.382 | cereal | No | 37 |
| S054 | Steep permanent | 8470 | 15 | 3413818.749 | 5915121.68 | rape | No | 63 |

**Table S2:** Characteristics of the plant species including colonization and dispersal traits. The seed longevity index ranged from short-lived seeds = 0 to long lived = 1 (Bekker et al. 1998). These data as well as data on species longevity (short=annual/biennial; long=perennial) were taken from the LEDA database (www.uni-oldenburg.de/en/landeco/research/leda/; Kleyer et al. 2011). Data on pollen dispersal (insect- or wind dispersal or selfing), seed dispersal (zoochory= Zoo, anemochory= anemo, hydrochory=hydro, hemerochory=hemero and autochory=autochor) and life strategies (C=Competitor, R=Ruderal, S=Stress-tolerant) were based on BIOLFLOR (http://www2.ufz.de/biolflor/), or Rothmaler (2005) and completed with the 3D Dispersal Diaspore Database (Hintze et. al 2013; www.seed-dispersal.info/terms-of-use.html) considering indices ranks > 0.5. SC = SC; SI = SI.

| **Species** | **Family** | **Type of kettle hole** | **Self-compatibility** | **Pollen vector** | **Seed dispersal** | **Life span** | **Seed bank longevity** | **Life strategies** |
| --- | --- | --- | --- | --- | --- | --- | --- | --- |
| *Acer platanoides* L. | Sapindaceae | Permanent | SI | insect | Anemo | long | 0 | C |
| *Acer pseudoplatanus* L. | Sapindaceae | Both | Both | insect | Anemo | long | 0 | C |
| *Achillea millefolium* L. | Asteraceae | Both | SI | insect | Zoo-Anemo-Auto | long | 0.1 | C |
| *Acorus calamus* L. | Acoraceae | Permanent | NA | NA | Hydro-Hemero | long | 0 | CS |
| *Aegopodium podagraria* L. | Apiaceae | Permanent | SC | insect | Zoo-Anemo | long | 0.26 | C |
| *Aethusa cynapium* L. | Apiaceae | Permanent | SC | insect/selfing | Anemo-Hemero | short | 0.97 | CR |
| *Agrostis capillaris* L. | Poaceae | Permanent | Both | wind | Anemo-Hydro | long | 0.52 | CSR |
| *Agrostis stolonifera* L. | Poaceae | Both | Both | wind | Zoo-Anemo-Hydro | long | 0.38 | CSR |
| *Alisma lanceolatum* With.^ab^ | Alismataceae | Ephemeral | SC | Insect/wind | Hydro | long | NA | CSR |
| *Alisma plantago-aquatica* L.^b^ | Alismataceae | Both | Both | Insect/wind | Zoo-Hydro | long | 0.84 | CSR |
| *Alliaria petiolata* (M. Bieb.) Cavara & Grande | Brassicaceae | Permanent | SC | insect/selfing | Zoo-Anemo-Hemero-Auto | short/long | 0.25 | CR |
| *Allium oleraceum* L. | Amaryllidaceae | Permanent | NA | insect | Hemero | long | 0 | CSR |
| *Allium vineale* L. | Amaryllidaceae | Permanent | NA | insect | Hemero | long | 0.08 | C |
| *Alopecurus aequalis* Sobol. | Poaceae | Both | SC | wind/selfing | Zoo-Anemo-Hydro | short/long | 0.67 | SR |
| *Alopecurus geniculatus* L. | Poaceae | Both | SC | wind | Zoo-Anemo-Hydro | short/long | 0.73 | CSR |
| *Alopecurus pratensis* L. | Poaceae | Permanent | SI | wind | Zoo-Anemo-Hydro | long | 0.08 | C |
| *Amaranthus retroflexus* L. | Amaranthaceae | Ephemeral | SC | wind/selfing | Zoo-Hemero | short | 0.79 | CR |
| *Anagallis arvensis* L. | Primulaceae | Both | SC | insect/selfing | Hemero | short | 0.82 | R |
| *Anchusa arvensis* (L.) M.Bieb. | Boraginaceae | Both | NA | insect/selfing | Zoo-Hemero-Auto | short/long | 0.27 | CR |
| *Anthriscus sylvestris* (L.) Hoffm. | Apiaceae | Permanent | SC | insect/selfing | Auto | short/long | 0.12 | C |
| *Apera spica-venti* (L.) P. Beauv. | Poaceae | Both | Both | wind | Zoo-Anemo-Hydro | short | 0.53 | CR |
| *Arctium lappa* L. | Asteraceae | Both | SC | insect/selfing | Zoo | short/long | 0.75 | C |
| *Arctium minus* (Hill) Bernh. | Asteraceae | Both | SC | insect/selfing | Zoo | short/long | 0.67 | C |
| *Arctium tomentosum* Mill. | Asteraceae | Both | SC | insect/selfing | Zoo-Anemo | short/long | 0 | C |
| *Armoracia rusticana* P. Gaertn., B.Mey. & Scherb. | Brassicaceae | Both | SI | insect/selfing | Hemero | long | 0 | C |
| *Arrhenatherum elatius* (L.) P. Beauv. ex J. Presl & C. Presl ^b^ | Poaceae | Permanent | Both | wind/selfing | Zoo-Anemo-Hydro | long | 0.06 | C |
| *Artemisia vulgaris* L. | Asteraceae | Both | NA | wind | Zoo-Hemero | long | 0.34 | C |
| *Athyrium filix-femina* (L.) Roth | Woodsiaceae | Permanent | Both | NA | Anemo | long | NA | CS |
| *Atriplex patula* L. | Amaranthaceae | Both | SC | insect/wind/selfing | Hydro-Hemero | short | 0.52 | CR |
| *Ballota nigra* L. | Lamiaceae | Permanent | SC | insect/selfing | Zoo | long | 0.63 | C |
| *Barbarea vulgaris* R.Br. | Brassicaceae | Permanent | Both | insect/selfing | Hydro-Hemero-Auto | short/long | 0.31 | CR |
| *Bidens tripartita* L. ^b^ | Asteraceae | Both | SC | insect/selfing | Zoo-Hydro | short | 0.4 | CR |
| *Bromus hordeaceus* L. | Poaceae | Both | SC | wind/selfing | Anemo | short/long | 0.13 | CR |
| *Bromus inermis* Leyss. | Poaceae | Permanent | Both | wind | Anemo-Hydro | long | 0 | C |
| *Bromus sterilis* L. | Poaceae | Both | SC | selfing | Zoo-Anemo | short/long | 0 | CR |
| *Bryonia alba* L. | Cucurbitaceae | Permanent | SC | insect | Zoo-Hemero | long | NA | C |
| *Calamagrostis canescens* (Weber) Roth | Poaceae | Permanent | SI | wind | Zoo-Anemo | long | 0.23 | CS |
| *Calamagrostis epigejos* (L.) Roth | Poaceae | Both | SI | wind | Zoo-Anemo-Hydro | long | 0.14 | C |
| *Capsella bursa-pastoris* (L.) Medik. | Brassicaceae | Both | SC | insect/selfing | Zoo-Hydro-Hemero-Auto | short | 0.65 | R |
| *Carduus crispus* Guirão ex Nyman | Asteraceae | Both | SC | insect/selfing | Anemo | short/long | 0.29 | CR |
| *Carex acuta* L. | Cyperaceae | Permanent | SI | wind | Anemo-Hydro | long | 0.26 | CS |
| *Carex acutiformis* Ehrh. | Cyperaceae | Permanent | SC | wind | Hydro | long | 0.09 | CS |
| *Carex hirta* L. | Cyperaceae | Permanent | SC | wind | Zoo-Anemo | long | 0.19 | C |
| *Carex pseudocyperus* L. | Cyperaceae | Permanent | SC | wind | Hydro | long | 0.5 | CS |
| *Carex remota* L. ^a^ | Cyperaceae | Permanent | SC | wind | Hydro | long | 0.83 | CS |
| *Carex riparia* Curtis | Cyperaceae | Both | SC | wind | Hydro | long | 0.15 | CS |
| *Carex vulpina* L. ^a^ | Cyperaceae | Permanent | SC | wind | Zoo-Anemo-Hydro | long | 0.83 | CSR |
| *Carpinus betulus* L. | Betulaceae | Ephemeral | NA | wind | Anemo-Other | long | 0 | C |
| *Centaurea cyanus* L. | Asteraceae | Both | NA | insect | Zoo-Anemo | short | 0.6 | CR |
| *Cerastium fontanum* supsp. *vulgare* (Hartm.) Greuter & Burdet | Caryophyllaceae | Permanent | SC | insect/selfing | Zoo-Hemero | short/long | 0.21 | CR |
| *Cerastium semidecandrum* L. | Caryophyllaceae | Permanent | SC | insect/selfing | Zoo-Anemo | short | 0.21 | R |
| *Prunus avium* L. | Rosaceae | Permanent | SI | insect/selfing | Zoo-Hemero | long | 0 | C |
| *Ceratophyllum demersum* L. ^a^ | Ceratophyllaceae | Both | SC | water | Hydro | long | 0 | NA |
| *Ceratophyllum submersum* L. | Ceratophyllaceae | Both | SC | water | Zoo-Hydro | long | NA | NA |
| *Chaerophyllum temulum* L. | Apiaceae | Permanent | SC | insect | Auto | long | NA | CR |
| *Chenopodium album* L. ^b^ | Amaranthaceae | Both | SC | wind | Zoo-Hydro-Hemero | short | 0.83 | CR |
| *Chenopodium polyspermum* L. | Amaranthaceae | Both | SC | wind | Zoo-Hemero | short | 0.76 | CR |
| *Chenopodium rubrum* L. | Amaranthaceae | Permanent | SC | insect/wind/selfing | Hydro-Hemero | long | 0.81 | CR |
| *Circaea lutetiana* L. | Onagraceae | Permanent | SC | insect/selfing | Zoo | long | 0 | CS |
| *Cirsium arvense* (L.) Scop. | Asteraceae | Both | SC | insect | Hydro | long | 0.2 | C |
| *Cirsium vulgare* (Savi) Ten. | Asteraceae | Both | SC | insect | Hydro | short/long | 0.2 | CR |
| *Clinopodium vulgare* L. | Lamiaceae | Permanent | SC | insect | Zoo-Auto | long | 0.44 | CS |
| *Convolvulus arvensis* L. | Convolvulaceae | Both | Both | insect/selfing | Zoo-Hemero | long | 0.1 | CR |
| *Conyza canadensis* (L.) Cronquist | Asteraceae | Both | NA | insect/selfing | Anemo | short/long | 0.5 | CR |
| *Crataegus monogyna* Jacq. | Rosaceae | Permanent | Both | insect/selfing | Zoo | long | 0.03 | C |
| *Cuscuta europaea* L. | Convolvulaceae | Permanent | SC | selfing | Zoo-Anemo | short | NA | NA |
| *Cynoglossum officinale* L. | Boraginaceae | Permanent | SC | insect/selfing | Zoo | short/long | 0.11 | CS |
| *Dactylis glomerata* L. | Poaceae | Both | Both | wind/selfing | Zoo-Anemo-Hydro | long | 0.15 | C |
| *Daucus carota* L. | Apiaceae | Both | SC | insect/selfing | Zoo-Hemero | short/long | 0.31 | CR |
| *Descurainia sophia* (L.) Webb ex Prantl | Brassicaceae | Permanent | SC | insect/selfing | Zoo-Anemo-Auto | short | 0.86 | CR |
| *Dipsacus fullonium* L. | Caprifoliaceae | Permanent | Both | insect/selfing | Hemero | short/long | 0.06 | CR |
| *Dryopteris filix-mas*(L.) Schott | Dryopteridaceae | Permanent | SC | NA | Anemo | long | NA | CS |
| *Echinochloa crus-galli* (L.) P.Beauv. ^b^ | Poaceae | Both | SC | wind/selfing | Zoo | short | 0.7 | CR |
| *Eleocharis palustris* (L.) Roem. & Schult. | Cyperaceae | Both | SC | wind | Zoo-Anemo | short/long | 0.22 | CS |
| *Elymus repens* (L.) Gould | Poaceae | Both | Both | wind | Zoo-Anemo | long | 0.07 | C |
| *Epilobium hirsutum* L. | Onagraceae | Both | SC | insect/selfing | Anemo | long | 0.64 | C |
| *Epilobium obscurum* (Schreb.) Schreb. ^a^ | Onagraceae | Permanent | SC | selfing | Anemo | long | 0.75 | CS |
| *Epilobium palustre* L. ^a^ | Onagraceae | Both | SC | insect/selfing | Anemo-Hydro | long | 0.25 | CSR |
| *Epilobium parviflorum* Schreb. | Onagraceae | Both | SC | insect/selfing | Anemo | long | 0.06 | CS |
| *Epilobium roseum* (Schreb.) Schreb. | Onagraceae | Permanent | SC | insect/selfing | Anemo | long | 0 | CS |
| *Epipactis helleborine* (L.) Crantz | Orchidaceae | Permanent | SC | insect/selfing | Zoo-Anemo | long | 0 | CSR |
| *Equisetum arvense* L. | Equisetaceae | Both | SC | NA | Anemo | long | NA | CR |
| *Equisetum palustre* L. | Equisetaceae | Both | SI | NA | Anemo-Hydro | long | NA | CSR |
| *Erysimum cheiranthoides* L. | Brassicaceae | Both | SC | insect/selfing | Anemo-Auto | short | 0.58 | CR |
| *Euonymus europaeus* L. | Celastraceae | Permanent | NA | insect | Zoo | long | 0 | C |
| *Euphorbia exigua* L. ^a^ | Euphorbiaceae | Ephemeral | SC | insect | Hemero | short/long | 0.85 | R |
| *Euphorbia helioscopia* L. | Euphorbiaceae | Both | SC | insect/selfing | Hemero-Auto | short | 0.63 | R |
| *Fagopyrum tataricum* (L.) Gaertn. | Polygonaceae | Permanent | SC | insect/wind/selfing | Hemero | short | NA | CR |
| *Falcaria vulgaris* Bernh. | Apiaceae | Permanent | SC | insect | Anemo-Hemero | short/long | 0 | CS |
| *Fallopia convolvulus* (L.) Á. Löve | Polygonaceae | Both | SC | insect/selfing | Zoo-Hemero | short | 0.63 | CR |
| *Festuca gigantea* (L.) Vill. | Poaceae | Permanent | SC | wind | Zoo-Anemo | long | 0.08 | CS |
| *Festuca rubra* L. | Poaceae | Permanent | Both | wind | Zoo-Anemo-Hydro | short/long | 0.14 | C |
| *Fraxinus excelsior* L. | Oleaceae | Permanent | Both | wind | Anemo | long | 0.02 | C |
| *Fumaria officinalis* L. ^b^ | Papaveraceae | Ephemeral | SC | insect/selfing | Zoo-Hemero | short | 0.61 | R |
| *Galeopsis bifida* Boenn. | Lamiaceae | Permanent | SC | insect/selfing | Zoo-Hydro | short | NA | CR |
| *Galeopsis speciosa* Mill. | Lamiaceae | Permanent | SC | insect/selfing | Zoo | short | 0 | CR |
| *Galeopsis tetrahit* L. | Lamiaceae | Both | SC | insect/selfing | Zoo | short | 0.45 | CR |
| *Galeopsis tetrahit x bifida* | Lamiaceae | Permanent | NA | NA | Zoo | short | NA | CR |
| *Galium aparine* L. | Rubiaceae | Both | SC | insect/selfing | Zoo-Hemero | short | 0.16 | CR |
| *Galium mollugo* L. | Rubiaceae | Permanent | Both | insect/selfing | Zoo | long | 0.1 | C |
| *Galium palustre* L. ^a^ | Rubiaceae | Permanent | SC | insect/selfing | Hydro | long | 0.18 | CS |
| *Genista tinctoria* L. | Fabaceae | Permanent | SI | insect | Auto | long | 0 | CS |
| *Geranium dissectum* L. ^a^ | Geraniaceae | Permanent | SC | insect/selfing | Hemero-Auto | short/long | 0.21 | CR |
| *Geranium pusillum* L. | Geraniaceae | Both | SC | insect/selfing | Zoo-Hemero | short/long | 0.38 | C |
| *Geranium robertianum* L. | Geraniaceae | Permanent | SC | insect/selfing | Zoo | short/long | 0.35 | CSR |
| *Geum urbanum* L. | Rosaceae | Both | SC | insect/selfing | Zoo | long | 0.05 | CSR |
| *Glechoma hederacea* L. | Lamiaceae | Permanent | Both | insect | Zoo | long | 0.2 | CSR |
| *Glyceria fluitans* (L.) R. Br. | Poaceae | Both | Both | wind | Zoo-Hydro | long | 0.61 | CS |
| *Glyceria maxima* (Hartm.) Holmb. | Poaceae | Permanent | NA | wind | Zoo-Hydro | long | 0.17 | CS |
| *Glyceria notata* Chevall. ^a^ | Poaceae | Both | SC | selfing | Zoo-Hydro | long | 1 | CS |
| *Gnaphalium sylvaticum* L. | Asteraceae | Permanent | SC | insect/selfing | Anemo | long | 0.71 | CSR |
| *Gnaphalium uliginosum* L. ^b^ | Asteraceae | Both | SC | insect/selfing | Zoo-Hydro | short | 0.89 | R |
| *Heracleum mantegazzianum* Sommier & Levier | Apiaceae | Ephemeral | SC | insect/selfing | Anemo-Hemero | short/long | 0 | C |
| *Heracleum sphondylium* L. | Apiaceae | Permanent | SC | insect/selfing | Zoo-Anemo-Hydro | short/long | 0.04 | C |
| *Holcus lanatus* L. | Poaceae | Permanent | Both | wind/selfing | Zoo-Anemo-Hydro | long | 0.51 | C |
| *Holcus mollis* L. | Poaceae | Permanent | Both | wind | Zoo-Anemo-Hydro | long | 0.03 | CSR |
| *Hottonia palustris* L. ^a^ | Primulaceae | Permanent | Both | insect | Zoo-Hydro | long | NA | NA |
| *Humulus lupulus* L. | Cannabaceae | Permanent | SI | wind | Anemo | long | 0.25 | C |
| *Hypericum perforatum* L. | Hypericaceae | Both | SC | insect/selfing | Zoo-Auto | long | 0.64 | C |
| *Impatiens glandulifera* Royle | Balsaminaceae | Permanent | SC | insect | Auto | short | 0 | CR |
| *Impatiens parviflora* DC. | Balsaminaceae | Permanent | SC | insect/selfing | Auto | short | 0 | SR |
| *Iris pseudacorus* L. | Iridaceae | Both | Both | insect | Zoo-Hydro-Hemero | long | 0 | CS |
| *Juncus articulatus* L. | Juncaceae | Both | SC | wind | Zoo-Auto | long | 0.91 | CSR |
| *Juncus bufonius* L. ^b^ | Juncaceae | Both | SC | wind/selfing | Zoo | short | 0.9 | R |
| *Juncus effusus* L. | Juncaceae | Both | SC | insect/wind/selfing | Zoo-Auto | long | 0.93 | C |
| *Juncus inflexus* L. ^a^ | Juncaceae | Permanent | SC | wind/selfing | Anemo | long | 0.63 | C |
| *Lactuca serriola* L. | Asteraceae | Both | SC | insect/selfing | Anemo | short/long | 0.21 | CR |
| *Lamium purpureum* L. | Lamiaceae | Both | NA | insect/selfing | Zoo-Anemo | short/long | 0.59 | R |
| *Lapsana communis* L. | Asteraceae | Permanent | SC | insect/selfing | Zoo-Hemero-Auto | short/long | 0.55 | CR |
| *Lathyrus pratensis* L. | Fabaceae | Permanent | Both | insect | Zoo-Auto | long | 0.05 | C |
| *Lemna gibba* L. | Araceae | Permanent | SC | wind/water/selfing/insect | Zoo-Hydro | NA | NA | NA |
| *Lemna minor* L. | Araceae | Both | Both | wind/water/selfing/insect | Zoo-Hydro | NA | 0 | NA |
| *Lemna trisulca* L. | Araceae | Both | SC | wind/water/selfing/insect | Zoo-Hydro | NA | 0 | NA |
| *Leontodon autumnalis* L. | Asteraceae | Permanent | Both | insect | Zoo-Anemo | long | 0.11 | CSR |
| *Lepidium ruderale* L. | Brassicaceae | Permanent | SC | selfing | Zoo-Anemo-Hemero | short | 0 | R |
| *Leucanthemum vulgare* (Vaill.) Lam. ^a^ | Asteraceae | Permanent | SC | insect/selfing | Zoo-Anemo-Hemero | long | 0.36 | C |
| *Lolium multiflorum* Lam. | Poaceae | Both | SI | wind | Zoo-Anemo-Hydro | short/long | 0.33 | C |
| *Lolium perenne* L. | Poaceae | Ephemeral | Both | wind | Zoo-Anemo-Hydro | short/long | 0.19 | C |
| *Lycopus europaeus* L. | Lamiaceae | Both | SC | insect | Zoo-Hydro | long | 0.19 | CS |
| *Lysimachia nummularia* L. | Primulaceae | Permanent | Both | insect | Hydro-Hemero | long | 0.08 | CSR |
| *Lysimachia vulgaris* L. | Primulaceae | Permanent | SI | insect/selfing | Hydro-Auto | long | 0.29 | CS |
| *Lythrum salicaria* L. | Lythraceae | Both | SI | insect/selfing | Zoo | long | 0.36 | CS |
| *Malus domestica* Borkh. | Rosaceae | Permanent | Both | insect | Zoo | long | 0 | C |
| *Matricaria discoidea* DC. | Asteraceae | Both | SC | insect/selfing | Zoo-Hemero | short | 0.86 | R |
| *Matricaria recutita* L. | Asteraceae | Both | NA | insect | Zoo-Hemero | short/long | 0.72 | R |
| *Medicago lupulina* L. | Fabaceae | Both | SC | insect/selfing | Zoo-Anemo-Hemero | short/long | 0.35 | CSR |
| *Melica uniflora* Retz. ^a^ | Poaceae | Permanent | NA | wind | Zoo | long | 0 | C |
| *Melilotus albus* Medik. | Fabaceae | Permanent | Both | insect/selfing | Zoo-Anemo | short/long | 0.05 | CR |
| *Mentha aquatica* L. | Lamiaceae | Permanent | SC | insect | Hydro-Auto | long | 0.51 | CS |
| *Mentha arvensis* L. | Lamiaceae | Both | SC | insect | Zoo-Hydro-Hemero | long | 0.28 | C |
| *Chaenorhinum minus* (L.) Lange | Plantaginaceae | Ephemeral | SC | selfing | Anemo | NA | NA | R |
| *Milium effusum* L. | Poaceae | Permanent | SC | wind/selfing | Zoo | long | 0.53 | CS |
| *Moehringia trinervia* (L.) Clairv. | Caryophyllaceae | Permanent | SC | insect/selfing | Zoo | short/long | 0.59 | CSR |
| *Myosotis arvensis* (L.) Hill | Boraginaceae | Both | SC | insect/selfing | Zoo-Hemero | short/long | 0.44 | R |
| *Myosurus minimus* L. ^a^ | Ranunculaceae | Ephemeral | SC | insect/selfing | Zoo-Anemo | short/long | 1 | CS |
| *Oenanthe aquatica* (L.) Poir. | Apiaceae | Both | SC | insect/selfing | Zoo-Hydro | short/long | 0 | R |
| *Papaver rhoeas* L. | Papaveraceae | Both | SI | insect | Anemo-Auto | short/long | 0.63 | CS |
| *Lythrum portula* (l.) D.A. Webb ^b^ | Lythraceae | Both | SC | selfing | Zoo-Hydro | NA | 1 | CSR |
| *Petasites hybridus* (L.) G. Gaertn., B. Mey. & Scherb. ^b^ | Asteraceae | Ephemeral | SI | insect | Zoo-Anemo-Hydro | long | 0 | CR |
| *Phacelia tanacetifolia* Benth. | Boraginaceae | Permanent | SC | insect/selfing | NA | short | NA | S |
| *Phalaris arundinacea* L. | Poaceae | Both | SI | wind/selfing | Zoo-Anemo-Hydro | long | 0.06 | CS |
| *Phleum pratense* L. | Poaceae | Both | Both | insect/wind/selfing | Zoo-Anemo | long | 0.2 | C |
| *Phragmites australis* (Cav.) Trin. ex Steud. | Poaceae | Permanent | Both | wind | Hydro | long | 0.02 | C |
| *Picris hieracioides* Sibth. & Sm. | Asteraceae | Both | SI | insect/selfing | Anemo | short/long | 0.18 | C |
| *Plantago lanceolata* L. | Plantaginaceae | Both | Both | insect/wind/selfing | Zoo-Auto | long | 0.24 | CS |
| *Plantago major* subsp. *intermedia* (Gilib.) Lange | Plantaginaceae | Both | SC | wind/selfing | NA | NA | NA | CSR |
| *Poa annua* L. | Poaceae | Both | SC | wind/selfing | Zoo-Anemo | short | 0.83 | S |
| *Poa nemoralis* L. | Poaceae | Permanent | SC | wind | Zoo-Anemo | long | 0.39 | CS |
| *Poa palustris* L. | Poaceae | Both | SC | wind | Zoo-Anemo-Hydro | long | 0.11 | C |
| *Poa trivialis* L. | Poaceae | Both | SC | wind | Zoo-Anemo-Hydro | NA | 0.59 | R |
| *Polygonum amphibium* L. | Polygonaceae | Both | SI | insect | Zoo-Hydro | long | 0 | R |
| *Polygonum aviculare* L. ^b^ | Polygonaceae | Both | SC | insect/selfing | Zoo-Hemero | short | 0.57 | CR |
| *Polygonum hydropiper* L. | Polygonaceae | Both | SC | selfing | Zoo-Hydro-Hemero | short | 0.4 | CS |
| *Polygonum lapathifolium* L. | Polygonaceae | Both | SC | insect/selfing | Zoo-Hydro-Hemero | short | 0.69 | R |
| *Polygonum minus* Huds. | Polygonaceae | Both | SC | insect/selfing | Hydro | short | 0.25 | CR |
| *Polygonum persicaria* L. | Polygonaceae | Both | NA | NA | Zoo-Hydro-Hemero | short | 0.69 | CS |
| *Populus tremula* L. | Salicaceae | Permanent | SI | wind | Anemo | long | 0 | CR |
| *Potamogeton natans* L. | Potamogetonaceae | Ephemeral | SC | wind | Zoo | NA | NA | C |
| *Potentilla anserina* L. | Rosaceae | Permanent | SI | insect | Zoo-Hydro-Hemero | long | 0.17 | NA |
| *Potentilla reptans* L. | Rosaceae | Both | NA | insect | Zoo-Hydro | long | 0.33 | S |
| *Prunus cerasifera* Ehrh. | Rosaceae | Permanent | Both | insect | Hemero | NA | 0 | CS |
| *Prunus domestica* L. | Rosaceae | Permanent | Both | insect/selfing | Hemero | long | 0 | CS |
| *Prunus spinosa* L. | Rosaceae | Permanent | NA | insect/selfing | Zoo-Hemero | long | 0 | C |
| *Quercus petraea* (Matt.) Liebl. | Fagaceae | Permanent | SI | wind | Other | long | 0 | CSR |
| *Quercus robur* L. | Fagaceae | Permanent | Both | wind | Other | long | 0 | C |
| *Ranunculus repens* L. | Ranunculaceae | Both | Both | insect/selfing | Zoo-Hemero-Auto | long | 0.54 | C |
| *Ranunculus sceleratus* L. ^b^ | Ranunculaceae | Both | SI | insect | Zoo-Hydro | short | 0.79 | SR |
| *Raphanus raphanistrum* L. | Brassicaceae | Permanent | SI | insect/selfing | Zoo-Anemo-Hydro-Hemero | short/long | 0.3 | CR |
| *Reynoutria sachalinensis* (F. Schmidt) Nakai | Polygonaceae | Permanent | SI | insect | Anemo-Hydro-Hemero | long | NA | CR |
| *Ribes uva-crispa* L. | Grossulariaceae | Permanent | NA | insect/selfing | Auto | long | 0 | C |
| *Elymus caninus* (L.) L. ^a^ | Poaceae | Permanent | SC | wind/selfing | Zoo-Anemo-Hydro | long | 0.67 | CS |
| *Rorippa amphibia* (L.) Besser | Brassicaceae | Both | SI | insect/selfing | Zoo-Hydro | long | 0.25 | CS |
| *Rorippa palustris* (L.) Besser ^b^ | Brassicaceae | Both | SC | insect/selfing | Zoo-Hydro | short/long | 0.63 | CR |
| *Rosa spec.* | Rosaceae | Permanent | NA | NA | Zoo-Hemero-Auto | long | 0.04 | C |
| *Rubus caesius* L. | Rosaceae | Permanent | SC | insect/selfing | Zoo | long | 0 | C |
| *Rubus idaeus* L. | Rosaceae | Permanent | SI | insect/selfing | Auto | long | 0.68 | C |
| *Rubus spec.* | Rosaceae | Permanent | NA | NA | Hemero-Auto | long | NA | C |
| *Rumex crispus* L. | Polygonaceae | Both | Both | wind | Zoo-Anemo | short/long | 0.3 | NA |
| *Rumex maritimus* L. ^b^ | Polygonaceae | Both | SC | wind/selfing | Zoo-Anemo-Hydro | short/long | 0.95 | SR |
| *Rumex obtusifolius* L. | Polygonaceae | Both | Both | wind | Zoo-Anemo-Hydro | long | 0.54 | SR |
| *Rumex patientia* L. | Polygonaceae | Both | NA | wind | NA | NA | 0 | C |
| *Rumex sanguineus* L. ^a^ | Polygonaceae | Both | NA | wind | Zoo | long | 0.83 | CS |
| *Rumex stenophyllus* Ledeb. | Polygonaceae | Ephemeral | NA | wind | NA | NA | NA | C |
| *Salix alba* L. ^a^ | Salicaceae | Both | SI | insect | Anemo-Hydro | long | 0.44 | R |
| *Salix caprea* L. | Salicaceae | Permanent | SI | insect | Anemo-Hydro | long | 0.02 | C |
| *Salix cinerea* L. | Salicaceae | Both | SI | insect | Anemo-Hydro | long | 0 | C |
| *Salix silesiaca* Willd. | Salicaceae | Permanent | SI | insect | Anemo-Hydro | long | NA | C |
| *Salix viminalis* L. | Salicaceae | Both | SI | insect | Anemo-Hydro | NA | 0 | SR |
| *Sambucus nigra* L. | Adoxaceae | Both | SC | insect/selfing | Hemero | long | 0.22 | C |
| *Schoenoplectus lacustris* (L.) Palla | Cyperaceae | Permanent | SC | Insect/wind | Zoo | NA | 0 | CSR |
| *Scrophularia nodosa* L. | Scrophulariaceae | Permanent | SC | insect | Auto | long | 0.9 | CS |
| *Scrophularia umbrosa* Dumort. | Scrophulariaceae | Permanent | NA | insect/selfing | Hydro-Auto | long | 1 | C |
| *Scutellaria galericulata* L. | Lamiaceae | Permanent | SC | insect/selfing | Hydro-Auto | long | 0 | CSR |
| *Senecio vulgaris* L. | Asteraceae | Ephemeral | Both | insect/selfing | Zoo-Hydro | short/long | 0.55 | C |
| *Setaria viridis* (L.) P. Beauv. | Poaceae | Ephemeral | SC | wind | Zoo | short | 0.77 | CS |
| *Silene latifolia* Poir. | Caryophyllaceae | Permanent | SC | insect | Hemero-Auto | NA | 0.44 | CR |
| *Silene noctiflora* L. ^a^ | Caryophyllaceae | Both | SC | insect/selfing | Hemero-Auto | short | 1 | R |
| *Sinapis arvensis* L. | Brassicaceae | Permanent | SI | insect/selfing | Zoo-Hemero-Auto | short | 0.83 | S |
| *Sisymbrium officinale* (L.) Scop. | Brassicaceae | Both | SC | insect/selfing | Hemero-Auto | short | 0.41 | R |
| *Solanum dulcamara* L. | Solanaceae | Both | SC | insect | Zoo | long | 0.22 | SR |
| *Sonchus arvensis* L. | Asteraceae | Both | SI | insect/selfing | Anemo | long | 0.78 | R |
| *Sonchus asper* (L.) Hill | Asteraceae | Both | SC | insect/selfing | Zoo-Anemo | short | 0.51 | CS |
| *Sonchus oleraceus* L. | Asteraceae | Permanent | SC | selfing | Zoo-Anemo | short | 0.75 | C |
| *Sorbus aucuparia* L. | Rosaceae | Permanent | Both | insect | Zoo-Other | long | 0.02 | C |
| *Sparganium emersum* Rehmann ^a^ | Typhaceae | Ephemeral | SC | wind | Anemo | NA | NA | C |
| *Sparganium erectum* L. | Typhaceae | Both | SC | Insect/wind | Zoo-Hydro | long | 0 | SR |
| *Spergularia rubra* (L.) J. Presl & C. Presl | Caryophyllaceae | Ephemeral | SC | insect/selfing | Zoo | short/long | 1 | SR |
| *Spirodela polyrhiza* (L.) Schleid. ^b^ | Araceae | Both | SC | wind/water/selfing/insect | Zoo-Hydro | NA | NA | C |
| *Stachys palustris* L. | Lamiaceae | Permanent | SC | insect/selfing | Hydro-Auto | long | 0.04 | NA |
| [*Stellaria aquatica* (L.) Scop.](http://www.theplantlist.org/tpl1.1/record/kew-2482222) ^b^ | Caryophyllaceae | Permanent | SC | insect/selfing | Zoo-Anemo-Hydro | long | 0 | C |
| *Stellaria media* (L.) Vill. | Caryophyllaceae | Both | Both | insect/selfing | Zoo-Hydro-Hemero | short/long | 0.71 | R |
| *Stellaria palustris* Ehrh. ex Retz. | Caryophyllaceae | Permanent | SC | insect/selfing | Hydro | long | 0.13 | CS |
| *Symphytum officinale* L. | Boraginaceae | Permanent | NA | insect/selfing | Zoo-Hydro-Hemero | long | 0 | C |
| *Tanacetum vulgare* L. | Asteraceae | Ephemeral | NA | insect | Zoo-Hydro-Hemero | long | 0.16 | CSR |
| *Taraxacum spec.* | Asteraceae | Both | NA | NA | Zoo-Anemo | NA | 0.26 | CS |
| *Thlaspi arvense* L. ^b^ | Brassicaceae | Both | SC | insect/selfing | Zoo-Anemo-Hemero-Auto | short | 0.87 | CS |
| *Torilis japonica* (Houtt.) DC. | Apiaceae | Permanent | SC | insect | Zoo | short/long | 0.47 | CSR |
| *Trifolium hybridum* L. | Fabaceae | Both | SI | insect | Zoo | long | 0.5 | R |
| *Trifolium incarnatum* L. | Fabaceae | Permanent | NA | insect/selfing | Zoo-Anemo | NA | 1 | R |
| *Trifolium pratense* L. | Fabaceae | Permanent | SI | insect | Zoo | long | 0.24 | SR |
| *Trifolium repens* L. | Fabaceae | Both | Both | insect | Zoo | long | 0.38 | C |
| *Tripleurospermum perforatum* (Mérat) Laínz | Asteraceae | Both | Both | insect | Zoo-Hemero | NA | 0.74 | CSR |
| *Typha latifolia* L. | Typhaceae | Both | SC | wind | Anemo-Hydro | long | 0.58 | CS |
| *Urtica dioica* L. ^b^ | Urticaceae | Both | SC | Insect/wind | Anemo-Hydro-Auto | long | 0.65 | R |
| *Veronica anagallis-aquatica* L. ^a^ | Plantaginaceae | Both | SC | insect/selfing | Zoo | long | 0.67 | R |
| *Veronica chamaedrys* L. | Plantaginaceae | Permanent | NA | selfing | Zoo-Hydro | long | 0.42 | S |
| *Veronica persica* Poir. L. | Plantaginaceae | Both | SC | insect/selfing | Hemero | short/long | 0.66 | CSR |
| *Veronica serpyllifolia* L. | Plantaginaceae | Ephemeral | SC | insect | Zoo | short/long | 0.71 | CS |
| *Vicia sativa subsp. nigra* (L.) Ehrh. | Fabaceae | Both | SC | insect/selfing | Auto | short/long | 0 | C |
| *Vicia cracca* L. | Fabaceae | Permanent | SC | insect | Zoo-Auto | long | 0.05 | C |
| *Vicia hirsuta* (L.) Gray | Fabaceae | Both | SC | insect/selfing | Zoo-Auto | short/long | 0.25 | CR |
| *Vicia tetrasperma* (L.) Schreb. | Fabaceae | Permanent | SC | insect/selfing | Zoo-Auto | short/long | 0.2 | CS |
| *Vicia villosa* Roth | Fabaceae | Both | NA | insect | Hemero | short/long | 0.18 | CSR |
| *Viola arvensis* Murray | Violaceae | Both | SC | insect/selfing | Auto | short | 0.53 | S |

^a^ Plant species present in the state red-list of Brandenburg (Ristow et al. 2006).

^b^ Plant species from seed banks were found.

**Table S3:** List of species with seeds were found in the seedbank in a subset of 20 kettle holes.

| Species | # individuals in flat ephemeral ponds | # individuals in steep permanent ponds |
| --- | --- | --- |
| *Alisma plantago-aquatica* L. | 1 | 23 |
| *Alisma lanceolatum* With. | 0 | 1 |
| *Arrhenatherum elatius* L. | 2 | 3 |
| *Bidens tripartita* L. | 112 | 57 |
| *Brassica napus* L. | 0 | 5 |
| *Brassica* spec. 1 | 8 | 16 |
| *Brassica* spec. 2 | 60 | 2 |
| *Brassica* spec. 3 | 0 | 13 |
| *Chenopodium album* L. | 335 | 21 |
| *Echinochloa curs-galli* (L.) P. Beauv. | 15 | 7 |
| *Epilobium* spec. | 49 | 6 |
| *Fumaria officinalis* L. | 5 | 10 |
| *Geranium* spec. | 0 | 5 |
| *Gnaphalium uliginosum* L. | 12 | 2 |
| *Juncus bufonius* L. | 2653 | 1255 |
| *Juncus* spec. 1 | 167 | 142 |
| *Juncus* spec. 2 | 34 | 99 |
| *Juncus* spec. 3 | 0 | 3 |
| *Lythrum portula* (L.) D.A.Webb | 64 | 0 |
| *Matricaria* spec. | 377 | 264 |
| *Myosotis* spec. | 0 | 1 |
| *Oenanthe* spec. | 0 | 73 |
| *Petasites hybridus* (L.) G. Gaerth, B. Mey. & Scherb | 77 | 0 |
| *Polygonum* spec. | 0 | 9 |
| *Polygonum aviculare* L. | 1868 | 198 |
| *Ranunculus sceleratus* L. | 28 | 135 |
| *Rorippa palustris* (L.) Besser | 153 | 32 |
| *Rumex maritimus* L. | 100 | 0 |
| *Spirodela polyrhiza* (L.) Schleid. | 27 | 53 |
| [*Stellaria aquatica* (L.) Scop.](http://www.theplantlist.org/tpl1.1/record/kew-2482222) | 134 | 41 |
| *Thlaspi arvense* L. | 0 | 41 |
| *Urtica dioica* L. | 104 | 901 |
| Unknown species x | 1 | 0 |
| Unknown species y | 146 | 31 |
| **Total # individuals** | **6532** | **3449** |
| **Average number** | **192.12** | **101.44** |
| **Standard Error** | **92.83** | **44.4** |
| **N species** | **25** | **30** |
| **N species only (Both=21)** | **4** | **9** |

**Table S4:** Detailed Generalized Linear Models with a quasi-poisson distribution selection based on Explanatory Deviance and qAIC of germination in relation with type of kettle hole and treatment for the seed bank experiment and for species number in relation with area (log) and degree of spatial isolation for the entire community and for wetlands specialized species.

**Seed bank experiment**

| **Model** | **glmID** | **ExplDev** | **adjExplDev** | **qAICc** | **dqAICc** | **df** | **weight** | **Model description** |
| --- | --- | --- | --- | --- | --- | --- | --- | --- |
| **1** | **m1qp** | **0.025556** | **-0.02858** | **222.35** | **0** | **2** | **0.463503** | **Germination ~ Type** |
| 2 | m2qp | 0.005269 | -0.04999 | 226.74 | 4.390183 | 2 | 0.05161 | Germination ~ Treatment |
| 3 | m3qp | 0.030825 | -0.0832 | 223.24 | 0.889648 | 3 | 0.297076 | Germination ~ Type + Treatment |
| 4 | m4qp | 0.030853 | -0.15086 | 225.27 | 2.920979 | 4 | 0.107589 | Germination ~ Type * Treatment |
| 5 | m5qp | 0 | 0 | 225.86 | 3.508039 | 1 | 0.080222 | Germination ~ 1 |

**Entire community**

| **Model** | **glmID** | **ExplDev** | **adjExplDev** | **qAICc** | **dqAICc** | **df** | **weight** | **Model description** |
| --- | --- | --- | --- | --- | --- | --- | --- | --- |
| 1 | m1qp | 0.0991 | 0.0786 | 222 | 54.5 | 2 | <0.001 | All_sp ~ Freq |
| 2 | m2qp | 0.2846 | 0.2684 | 200.5 | 33 | 2 | <0.001 | All_sp ~ Area_ha_log |
| 3 | m3qp | 0.2461 | 0.2289 | 205 | 37.5 | 2 | <0.001 | All_sp ~ Type |
| 4 | m4qp | 0.3454 | 0.3150 | 195.9 | 28.4 | 3 | <0.001 | All_sp ~ Area_ha_log + Freq |
| 5 | m5qp | 0.3488 | 0.3185 | 195.5 | 28 | 3 | <0.001 | All_sp ~ Freq + Type |
| 6 | m6qp | 0.4695 | 0.4448 | 181.5 | 14 | 3 | <0.001 | All_sp ~ Area_ha_log + Type |
| 7 | m7qp | 0.3679 | 0.3228 | 195.8 | 28.3 | 4 | <0.001 | All_sp ~ Area_ha_log * Freq |
| 8 | m8qp | 0.4548 | 0.4159 | 185.7 | 18.3 | 4 | <0.001 | All_sp ~ Freq * Type |
| 9 | m9qp | 0.4695 | 0.4316 | 184 | 16.6 | 4 | <0.001 | All_sp ~ Area_ha_log * Type |
| 10 | m10qp | 0.5365 | 0.5034 | 176.3 | 8.8 | 4 | 0.0094 | All_sp ~ Area_ha_log + Freq + Type |
| 11 | m11qp | 0.5365 | 0.4913 | 178.9 | 11.5 | 5 | 0.0025 | All_sp ~ Area_ha_log + Freq + Area_ha_log * Type |
| **12** | **m12qp** | **0.6356** | **0.6000** | **167.5** | **0** | **5** | **0.7654** | **All_sp ~ Area_ha_log + Freq * Type** |
| 13 | m13qp | 0.5551 | 0.5117 | 176.8 | 9.3 | 5 | 0.0073 | All_sp ~ Area_ha_log * Freq * Type |
| 14 | m14qp | 0.6632 | 0.6113 | 170 | 2.5 | 7 | 0.2145 | All_sp ~ Area_ha_log * Freq + Area * Type + Freq * Type |
| 15 | m15qp | 0.0000 | 0.0000 | 231.1 | 63.7 | 1 | <0.001 | All_sp ~ 1 |

**Wetland community**

| **Model** | **glmID** | **ExplDev** | **adjExplDev** | **qAICc** | **dqAICc** | **df** | **weight** | **Model description** |
| --- | --- | --- | --- | --- | --- | --- | --- | --- |
| 1 | sp1qp | 0.0749 | 0.0539 | 183.79 | 30.85 | 2 | 0.0000 | Wet_sp ~ Freq |
| 2 | sp2qp | 0.2872 | 0.2710 | 165.13 | 12.19 | 2 | 0.0021 | Wet _sp ~ Area_ha_log |
| 3 | sp3qp | 0.0597 | 0.0384 | 185.12 | 32.18 | 2 | 0.0000 | Wet _sp ~ Type |
| 4 | sp4qp | 0.3298 | 0.2986 | 163.79 | 10.85 | 3 | 0.0041 | Wet _sp ~ Area_ha_log + Freq |
| 5 | sp5qp | 0.1363 | 0.0962 | 180.80 | 27.86 | 3 | 0.0000 | Wet _sp ~ Freq + Type |
| 6 | sp6qp | 0.3174 | 0.2857 | 164.88 | 11.94 | 3 | 0.0024 | Wet _sp ~ Area_ha_log + Type |
| 7 | sp7qp | 0.3305 | 0.2827 | 166.26 | 13.32 | 4 | 0.0012 | Wet _sp ~ Area_ha_log * Freq |
| 8 | sp8qp | 0.3000 | 0.2500 | 168.94 | 16.00 | 4 | 0.0003 | Wet _sp ~ Freq * Type |
| 9 | sp9qp | 0.3224 | 0.2740 | 166.97 | 14.03 | 4 | 0.0008 | Wet _sp ~ Area_ha_log * Type |
| 10 | sp10qp | 0.3622 | 0.3166 | 163.47 | 10.53 | 4 | 0.0048 | Wet _sp ~ Area_ha_log + Freq + Type |
| 11 | sp11qp | 0.3670 | 0.3053 | 165.70 | 12.76 | 5 | 0.0016 | Wet _sp ~ Area_ha_log + Freq + Area_ha_log * Type |
| **12** | **sp12qp** | **0.5122** | **0.4646** | **152.94** | **0.00** | **5** | **0.9229** | **Wet _sp ~ Area_ha_log + Freq * Type** |
| 13 | sp13qp | 0.3626 | 0.3004 | 166.09 | 13.15 | 5 | 0.0013 | Wet _sp ~ Area_ha_log * Freq * Type |
| 14 | sp14qp | 0.5147 | 0.4401 | 158.45 | 5.51 | 7 | 0.0586 | Wet _sp ~ Area_ha_log * Freq + Area * Type + Freq * Type |
| 15 | sp15qp | 0.0000 | 0.0000 | 188.08 | 35.14 | 1 | 0.0000 | Wet _sp ~ 1 |

**Best Models**

**Seed bank: m1qp**

glm.nb (Germination ~ Type, data=Seed Bank Data, family=”quasipoisson”))

**All species: m12qp**

glm.nb (All_sp ~ Area_ha_log + Freq * Type, data=SITES, family=”quasipoisson”))

**Wetland species: sp12qp**

glm.nb (Wet_sp ~ Area _ha_log + Freq * Type, data=SITES, family=”quasipoisson”))

**Table S5:** Best fitted Linear Models and ANOVAs of colonization and dispersal traits.

| **TRAIT** | **BEST FITTED MODEL** |  | **SIGNIFICANT FACTORS** |  | **INTERACTIONS** |
| --- | --- | --- | --- | --- | --- |
| **Seed Longevity Index** | SLI.m08 <- lm(SLI_mean~Type_of_kettle_hole + Tree_presence + NEAR_X + Type_of_kettle_hole*Tree_presence + Type_of_kettle_hole*NEAR_X+ Tree_presence*NEAR_X + Type_of_kettle_hole*Tree_presence*NEAR_X) |  | **Type_of_kettle_hole ***** | Tree_presence** | NEAR_X** |
| R^2^ = 0.70 |  | F _1,40_ | 91.3155 | 8.6062 | 9.8589 |
|  |  | p | 7.04E-12 | 0.005523 | 0.003173 |
| **Short-lived** | Short.m08 <- lm(Per_sp_Short~Type_of_kettle_hole + Tree_presence + N_sp + Type_of_kettle_hole*Tree_presence + Type_of_kettle_hole*N_sp + Tree_presence*NEAR_X + Type_of_kettle_hole*Tree_presence*N_sp) |  | **Type_of_kettle_hole***** | Tree_presence * | Type_of_kettle_hole:N_sp . |
| R^2^ = 0.65 |  | F _1,32_ | 46.9606 | 6.4047 | 3.8207 |
|  |  | p | 3.89E-08 | 0.01564 | 0.05801 |
| **Long-lived** | Long.m08 <- lm(Per_sp_Long~Type_of_kettle_hole + Tree_presence + N_sp + Type_of_kettle_hole*Tree_presence + Type_of_kettle_hole*N_sp+ Tree_presence*NEAR_X + Type_of_kettle_hole*Tree_presence*N_sp) |  | **Type_of_kettle_hole ***** | Tree_presence * | N_sp * |
| R^2^ = 0.57 |  | F _1,38_ | 61.3397 | 5.9637 | 5.2972 |
|  |  | p | 1.90E-09 | 0.01937 | 0.02694 |
| **SC** | SC.m06 <- lm(Per_sp_SC~Type_of_kettle_hole + N_sp) |  | Type_of_kettle_hole | N_sp * |  |
| R^2^ = 0.19 |  | F _1,43_ | 0 | 5.5361 |  |
|  |  | p | 0.99567 | 0.02327 |  |
| **SI** | SI.m04 <- lm(Per_sp_SI~Type_of_kettle_hole + Tree_presence) |  | **Type_of_kettle_hole ***** | Tree_presence * |  |
| R^2^ = 0.26 |  | F _1,43_ | 15.2226 | 5.5381 |  |
|  |  | p | 0.0003312 | 0.0232493 |  |
|  |  |  |  |  |  |
| **Zoophily** | In.pol.m08 <- lm(Per_sp_Zoophily~Type_of_kettle_hole + Tree_presence + N_sp + Type_of_kettle_hole*Tree_presence + Type_of_kettle_hole*N_sp + Tree_presence*NEAR_X + Type_of_kettle_hole*Tree_presence*N_sp) |  | **Type_of_kettle_hole **** | N_sp ** | Type_of_kettle_hole:N_sp . |
| R^2^ = 0.31 |  | F _1,38_ | 10.5447 | 8.6824 | 3.3277 |
|  |  | p | 0.002437 | 0.005464 | 0.075993 |
| **Anemophily** | Wind.pol.m08 <- lm(Per_sp_Anemophily~Type_of_kettle_hole + Tree_presence + N_sp+ Type_of_kettle_hole*Tree_presence + Type_of_kettle_hole*N_sp + Tree_presence*NEAR_X + Type_of_kettle_hole*Tree_presence*N_sp) |  | Type_of_kettle_hole | NEAR_X ** | N_sp * |
|  |  | F _1,38_ | 0.0766 | 4.9158 | 9.123 |
| R^2^ = 0.17 |  | p | 0.783406 | 0.006835 | 0.032669 |
| **Hydrophily** | Water.pol.m08 <- lm(Per_sp_Hydrophily~Type_of_kettle_hole + Tree_presence + N_sp + Type_of_kettle_hole*Tree_presence + Type_of_kettle_hole*N_sp + Tree_presence*NEAR_X + Type_of_kettle_hole*Tree_presence*N_sp) |  | **Type_of_kettle_hole **** | NEAR_X * | Type_of_kettle_hole:N_sp * |
|  |  | F _1,38_ | 11.5819 | 4.6384 | 4.7823 |
| R^2^ = 0.24 |  | p | 0.001582 | 0.037672 | 0.034979 |
| **Selfing** | Other.pol.m02 <- lm(Per_sp_Other_pol~Tree_presence) |  | Type_of_kettle_hole |  |  |
| R^2^ = 0.02 |  | F _1,38_ | 1.6973 |  |  |
|  |  | p | 0.1994 |  |  |
| **Zoochory** | In.dis.m08 <- lm(Per_sp_Zoochory~Type_of_kettle_hole + Tree_presence + N_sp + Type_of_kettle_hole*Tree_presence + Type_of_kettle_hole*N_sp + Tree_presence*NEAR_X +Type_of_kettle_hole*Tree_presence*N_sp) |  | **Type_of_kettle_hole **** | NEAR_X ** |  |
| R^2^ = 0.29 |  | F _1,38_ | 10.7986 | 7.321 |  |
|  |  | p | 0.00219 | 0.01015 |  |
| **Anemochory** | Wind.dis.m07 <- lm(Per_sp_Anemochory~Type_of_kettle_hole * N_sp) |  | **Type_of_kettle_hole ***** |  | Type_of_kettle_hole:N_sp * |
| R^2^ = 0.37 |  | F _1,38_ | 23.213 |  | 4.8462 |
|  |  | p | 1.92E-05 |  | 0.03325 |
| **Hydrochory** | Water.dis.m08 <- lm(Per_sp_Hydrochory~Type_of_kettle_hole + Tree_presence + N_sp +Type_of_kettle_hole*Tree_presence + Type_of_kettle_hole*N_sp + Tree_presence*NEAR_X + Type_of_kettle_hole*Tree_presence*N_sp) |  | Type_of_kettle_hole | NEAR_X *** |  |
| R^2^ = 0.25 |  | F _1,38_ | 0.076 | 17.8137 |  |
|  |  | p | 0.784276 | 0.000146 |  |
| **Hemerochory** | Hemero.dis.m07 <- lm(Per_sp_Hemerochory~Type_of_kettle_hole * N_sp) |  | **Type_of_kettle_hole ***** |  |  |
| R^2^ = 0.28 |  | F _1,42_ | 16.5826 |  |  |
|  |  | p | 0.0002017 |  |  |
| **Autochory** | Auto.dis.m08 <- lm(Per_sp_Autochory~Type_of_kettle_hole + Tree_presence + N_sp + Type_of_kettle_hole*Tree_presence + Type_of_kettle_hole*N_sp + Tree_presence*NEAR_X + Type_of_kettle_hole*Tree_presence*N_sp) |  | **Type_of_kettle_hole ***** | NEAR_X * |  |
| R^2^ = 0.35 |  | F _1,38_ | 20.7001 | 5.769 |  |
|  |  | p | 5.36E-05 | 0.02131 |  |

**SUPPLEMENTARY FIGURES**

**Fig. S1:** Soil analysis of a subset of 20 kettle holes. Comparison pH between flat ephemeral and steep permanent kettle holes (F_1,18_ = 3.71 *P* = 0.069).

**Fig. S2:** Additional colonization and dispersal plant traits. Percentage of SI (A), hydrophilous (B), autochorous (C) and long-lived (D) species differ significantly (A: F_1,43_ = 15.22 *P* < 0.001 ; B: F_1,38_ = 11.58 *P* < 0.01; C: F_1,38_ = 20.70 *P* < 0.001; D: F_1,38_ = 61.33 *P* < 0.001 between flat ephemeral and steep permanent kettle holes.
